# Urban Safety: An Image-Processing and Deep-Learning-Based Intelligent Traffic Management and Control System

**DOI:** 10.3390/s21227705

**Published:** 2021-11-19

**Authors:** Selim Reza, Hugo S. Oliveira, José J. M. Machado, João Manuel R. S. Tavares

**Affiliations:** 1 Faculdade de Engenharia, Universidade do Porto, Rua Dr. Roberto Frias, s/n, 4200-465 Porto, Portugal; up202003355@edu.fe.up.pt (S.R.); hmso.lsa@gmail.com (H.S.O.); 2 Departamento de Engenharia Mecânica, Faculdade de Engenharia, Universidade do Porto, Rua Dr. Roberto Frias, s/n, 4200-465 Porto, Portugal; jjmm@fe.up.pt

**Keywords:** intelligent traffic management, traffic forecasting, traffic signal control, image processing, deep learning, machine vision

## Abstract

With the rapid growth and development of cities, Intelligent Traffic Management and Control (ITMC) is becoming a fundamental component to address the challenges of modern urban traffic management, where a wide range of daily problems need to be addressed in a prompt and expedited manner. Issues such as unpredictable traffic dynamics, resource constraints, and abnormal events pose difficulties to city managers. ITMC aims to increase the efficiency of traffic management by minimizing the odds of traffic problems, by providing real-time traffic state forecasts to better schedule the intersection signal controls. Reliable implementations of ITMC improve the safety of inhabitants and the quality of life, leading to economic growth. In recent years, researchers have proposed different solutions to address specific problems concerning traffic management, ranging from image-processing and deep-learning techniques to forecasting the traffic state and deriving policies to control intersection signals. This review article studies the primary public datasets helpful in developing models to address the identified problems, complemented with a deep analysis of the works related to traffic state forecast and intersection-signal-control models. Our analysis found that deep-learning-based approaches for short-term traffic state forecast and multi-intersection signal control showed reasonable results, but lacked robustness for unusual scenarios, particularly during oversaturated situations, which can be resolved by explicitly addressing these cases, potentially leading to significant improvements of the systems overall. However, there is arguably a long path until these models can be used safely and effectively in real-world scenarios.

## 1. Introduction

Urban transportation is considered the lifeblood of the world’s economy, with a rapid increase of all sorts of vehicles and a stably increasing population in need of mobility, posing challenges to cities, with one of the major problems being the increase of traffic and the associated issues. According to the World Health Organization (https://www.who.int/publications/i/item/9789241565684, Last accessed on 16 November 2021), each year, 1.35 million people are killed and 20 million are wounded on roadways around the world. Road crash injuries are estimated to be the eighth leading cause of death globally, with an estimated cost among the fatal and wounded victims of approximately USD 1.8 trillion from 2015–2030, equivalent to a yearly tax of 0.12% on global GDP (Chen et al. [1]). Furthermore, according to INRIX (https://inrix.com/press-releases/2019-traffic-scorecard-us/, Last accessed on 16 November 2021), traffic congestion cost the U.S. economy nearly USD 88 billion in 2019 alone.

Intelligent Traffic Management and Control (ITMC) systems have emerged as a vital element of traffic management solutions, with the research community developing mechanisms to increase their accuracy, efficiency, and effectiveness. Traffic state forecast and intersection signal control are two main components that ITMC incorporates. Commonly, two genres of methods can be found, with the first component based on statistical methods and data-driven approaches, enabling the formulation hypotheses and the derivation of assumptions in a macroscopic and microscopic perspective for traffic flow. However, these approaches cannot handle unstable traffic conditions and complex road settings (Elhenawy and Rakha [2]). To overcome the nonlinearity of traffic, data-driven approaches, such a Support Vector Machines (SVMs), K-Nearest Neighbors (KNNs), Bayesian methods, and Neural Networks (NNs), enable one to overcome the limitations of statistical methods with promising results (Huang et al. [3]). However, for a model to achieve a good performance, a large amount of time series data is required, with the efficiency largely depending on how much a model can capture the spatial–temporal features of the traffic states. Moreover, corrupted or missing data pose difficulties for models, limiting the capacity to provide a useful and reliable forecasting result. Recently, deep-learning-based methods have addressed some of these limitations due to their ability to process large amounts of data efficiently and to capture hidden or unknown traffic dynamics (Bao et al. [4]).

On the other hand, efficient intersection traffic signal control, particularly in oversaturated conditions, requires actions to be taken based on the current traffic dynamic variables of the corresponding and neighboring intersections by the proper implementation of the policies. The most widely used method to tackle this problem is the FT controllers, which use historical data to determine the appropriate timing of traffic signals. However, this approach cannot meet the current traffic stochastic demands and handle unexpected traffic situations (Osorio and Wang [5]). Due to the limitations of the Fixed Time (FT) controllers, Webster’s method was introduced, where inductive detectors are employed to observe the actual traffic conditions and efficiently extend or terminate the green signal time by measuring the gap between vehicles. However, accumulative information is neglected, reducing the overall performance (Eriskin et al. [6]).

The Sydney Coordinated Adaptive Traffic System (SCAT) (Sims and Dobinson [7]) and Split, Cycle, and Offset Optimization Technique (SCOOT) (Hunt et al. [8]) adopt adaptive systems to suppress the drawbacks of the previous methods by gathering the data of the traffic flow in real-time at each intersection to control the timing of traffic lights effectively. The SCAT systems count vehicles at each stop line to gather traffic information, and the SCOOT applies a set of advanced detectors located upstream of the stop line. Using these detectors, the SCOOT provides a higher resolution of the current traffic conditions, such as traffic flow and the number of cars in the queue before they reach the stop line. The SCAT and SCOOT both use centralized control schemes, with systems being run locally, and the coordination between intersections is achieved by communication among the neighbors. For example, when an intersection releases several vehicles, it informs the next intersection about the time and number of vehicles to expect at a particular time. However, the performance of such methods heavily depends on the detector’s position and reliability. Recently, deep-learning models have been applied to self-adaptive traffic signal control, exhibiting substantially better performance in terms of accuracy and robustness (Bouktif et al. [9]).

**Figure 1:**
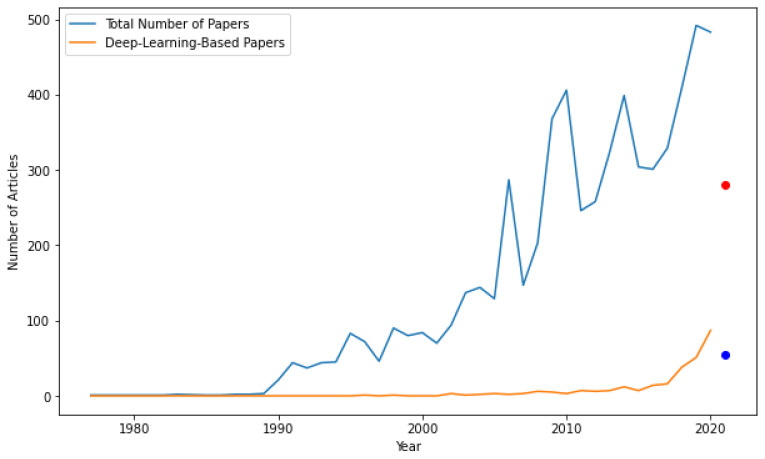
Number of published documents on ITMC in (1977–2021) found in the Scopus database illustrated both as a whole and as image-processing or deep-learning-based approaches (the dots represent the documents published in the year 2021 until July 2021).

The success of image-processing and the associated deep-learning technologies in ITMC in comparison with statistical methods can be realized from Figure 1, not only in terms of the quantity of articles published, but also in the quality of the forums in which they are published. In 2020, in the Scopus database, out of 483 published articles, 87 were based on deep-learning or image-processing methods, and they showed much better performances compared to state-of-the-art methods.

This review focuses on image-processing and deep-learning-based approaches to ITMC. Although there is a considerable number of relevant articles on intelligent transportation/traffic management and control (Nagy et al. [10], Pasquale et al. [11], Mirchandani et al. [12]), to the best of the authors’ knowledge, there are a limited number of works on ITMC based on image-processing and deep-learning-based. Within the literature, the new emerging image-processing and deep-learning techniques are at an early stage of development, but with an increasing number of relevant implementations among the research community. In the year 2011, there were in total 246 documents published on the Scopus database, whereas in 2020, that amounted to almost double, reaching 483, as summarized in Figure 1. Therefore, this study is valuable both for machine-learning and ITMC researchers and decision-makers, who could identify the potential advantages of ITMC in their practice.

The structure of this article as follows: Section 2 presents the methodologies used to identify and select the documents to be analyzed. Section 3 indicates different traffic state prediction/forecasting approaches and models with their corresponding structure, limitations, and performances. Section 4 is devoted to intersection-traffic-signal-control methods/policies, with a primary focus on their limitations and performances. Both in Section 3 and Section 4, particular emphasis is given to image-processing and deep-learning-based approaches, including a brief overview of commonly employed methods. In Section 5, the developed search of this review is described and complemented with significant research challenges found in the literature. Finally, Section 6 provides insights regarding the objectives drawn and points out the main conclusions.

## 2. Methodology of the Systematic Review

This section describes the research methodology used to locate, gather, and appraise the state-of-the-art works under study. The main requirement was to sort out the important recent works on intelligent traffic-management-and-control methods/systems based on image-processing and deep-learning approaches. The following complementary questions were considered:

- Which task of ITMC was addressed?
- Which dataset was used? Was it tested on different datasets?
- Which architecture/optimizer was utilized (developed or adapted)?
- What metrics were used for evaluation?
- Is the approach adopted or developed able to achieve real-time performance?
- For intersection-signaling schemes, what type of simulation environments (microscopic or macroscopic) were utilized? How were the policies evaluated?

### 2.1. Selection Criteria

The selection of the articles followed these criteria: (i) The studies should focus on intelligent traffic-management-and-control methods/systems based on the following approaches: image-processing and deep-learning techniques. The studies must identify problems, potential solutions, novelties, and limitations from which recommendations can be established. (ii) The studies should be peer-reviewed studies (research articles and literature reviews), best practices manuals (existing guidelines for ITMC), or policies. (iii) The research studies should include quantitative or qualitative research methods. (iv) All studies should be in English.

### 2.2. Databases and Search Steps

This literature review was conducted from March to July 2021 using Scopus, Science Direct, and Google Scholar. The authors extended the search to Google Scholar to include policies and best practices manuals. The search was performed with the following keywords in various combinations: “intelligent transportation”, “intelligent traffic management and control”, “image processing and deep learning-based intelligent traffic management and control”, “short-term traffic forecasting”, “image processing and deep learning-based short-term traffic forecasting”, “intersection traffic signal control”, “image processing and deep learning-based intersection traffic signal control”.

**Figure 2:**
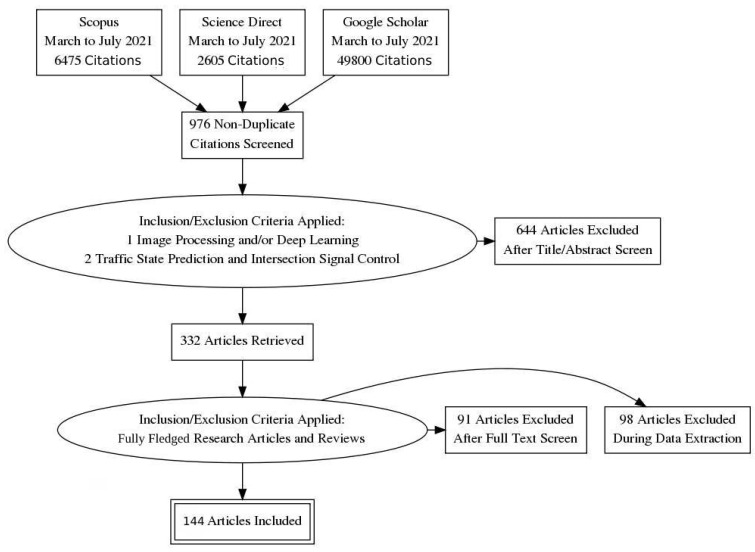
Adopted articles’ selection process on different databases illustrated according to the PRISMA diagram.

In the first step, we selected studies addressing the keywords: “intelligent transportation”, “intelligent traffic management and control”, “short-term traffic forecasting”, and “intersection traffic signal control” in various combinations. As a refinement step, we excluded duplicated articles and focused on image-processing and deep-learning approaches. The remaining articles were analyzed based on their titles and abstracts, with 332 articles retrieved. The 144 fully fledged research articles and reviews were sorted out to carry out in-depth studies through a complete reading of each document. We assessed the articles through the criteria that they should contain one of the following aspects: (1) research with actual users through qualitative or quantitative methods and the main method proposed and results obtained should be fully described; (2) specific guidelines or recommendations relating to the architecture, optimization, and metrics; (3) a review of existing literature regarding ITMC, as well as available policies that could contribute to the signaling schemes. In the final step, data from each document were organized in terms of type of study, primary focus, datasets used, adopted performance metrics, and limitations. Figure 2 illustrates these processes according to a PRISMA diagram.

## 3. Traffic State Prediction

**Figure 3:**
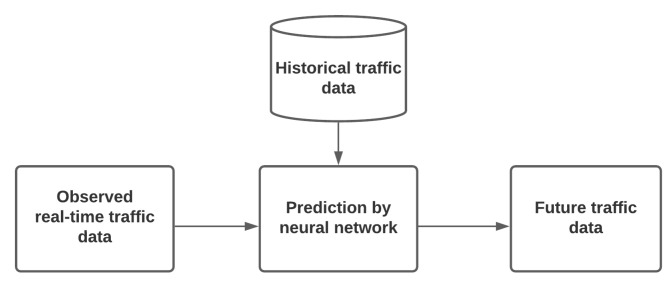
General block diagram of an NN-based traffic state prediction model (adapted from Do et al. [13]).

Traffic state prediction aims to forecast future traffic variables such as flow or speed in the road network based on historical or observed traffic data and other supporting information relevant to the demand. Most of the models used for forecasts found in the literature deal with parametric or nonparametric approaches. The most popular parametric approaches are the Autoregressive Integrated Moving Average (ARIMA) models. Among the nonparametric techniques, various models have been proposed, such as NNs, SVMs for regression, and KNNs. Figure 3 represents the basic building blocks of an NN-based traffic state prediction model.

### 3.1. Autoregressive Integrated Moving Average

One of the most used and classical models for time series forecast is ARIMA (Box et al. [14]), which is based on the principle that future time series values can be generated from a linear function of past observations and white noise terms. The main advantage of ARIMA is its flexibility in following data patterns and higher forecast accuracy in the short term (Irhami and Farizal [15]). However, it requires noise-free datasets for model construction and has limitations in capturing nonlinear features.

### 3.2. Support Vector Machines

Contrary to ARIMA, SVMs can handle nonlinear and high-dimensional problems. An SVM-based classifier tries to maximize the hyperplane separation between two classes by solving a linearly constrained quadratic programming problem. It is robust to overfitting while providing high generalization performance (Li and Xu [16] Mingheng et al. [17]). However, the SVM models perform better in forecasting medium-duration incident cases than high-duration incident cases (Yu et al. [18]).

### 3.3. K-Nearest Neighbors

KNN is a data-driven model, being extremely sensitive to the data quality. Nevertheless, KNN is able to forecast traffic state by exploring the correlation among the data as instance-based learning, avoiding searching in all historical data. For the short-term traffic state prediction under special events, KNN has the potentiality to find the most similar historical patterns and ignore other dissimilar ones of the datasets. However, in common with most other traditional machine-learning approaches, KNN faces the curse of dimensionality problem in network-wide traffic prediction (Yu et al. [19]).

### 3.4. Neural Networks

Within the literature, NN models used for traffic state forecast explore the use of Multilayer Feedforward Neural Networks (MLFNNs), Radial Basis Function Neural Networks (RBFNNs), Recurrent Neural Networks (RNNs), Convolutional Neural Networks (CNNs), Deep Belief Networks (DBNs), Wavelet Neural Networks (WNN)s, and Fuzzy Neural Networks (FNNs).

#### 3.4.1. Multilayer Feedforward Neural Networks

The MLFNN is a simple feedforward NN consisting of a layer of input units, one or more hidden units, and one layer of output units. The most pioneering contribution of short-term traffic forecasting using MLFNNs can be found in the works of Smith and Demetsky [20], Gilmore and Abe [21], Florio and Mussone [22], and Dougherty and Cobbett [23].

Smith and Demetsky proposed a simple MLFNN for short-term volume prediction with one hidden layer, trained using real-world data (open data at VDOT https://www.virginiaroads.org/datasets/traffic-volume/explore, Last accessed on 16 November 2021), which exhibited a lower performance compared with the nearest-neighbor model. Gilmore and Abe improved the accuracy by employing two hidden layers, taking into consideration training and simulation time to increase the accuracy of the works led by Florio and Mussone, with the use of three hidden layers and preprocessed training data to mitigate significant training time problems and increase the accuracy. Dougherty and Cobbett trained an MLFNN with one hidden layer to forecast short-term traffic flow, speed, and occupancy space. The results showed that speed forecast was much less successful, although flow and occupancy forecasts exhibited promising results. Capturing both spatial and temporal features of traffic states and the usage of a correction mechanism can mitigate the problems as identified by Polson and Sokolov [24] and Huang et al. [3].

Although the accuracy was not very promising, attempts were also made to model and forecast network-wide traffic using MLFNNs (Sun et al. [25], Elhenawy and Rakha [2]). Sun et al. combined Graphical Lasso (GL) with an NN for a multilink prediction model. Elhenawy and Rakha proposed a much more accurate and robust data-driven approach by considering current traffic state data, weather conditions, visibility levels, and seasonal predictors. Moreover, their work was a milestone for the identification of traffic problems up to 2 h in advance, when compared to Kumar et al. [26], whose work was only able to extend the time horizon to a maximum of 15 min.

MLFNN-optimization strategies were also found during the studies; for example, Vlahogianni et al. [27] proposed a genetic-algorithm-based, structural-optimization strategy to help in both the proper data representation with temporal and spatial features, as well as inappropriate structure selection. Table 1 summarizes the works using MLFNNs and their focus, limitations, and performances.

Due to their capabilities of modeling nonlinear functions with a simple architecture, MLFNNs have been extensively used in traffic state prediction. However, these models have some limitations in the exploration of more complex data correlations.

#### 3.4.2. Radial Basis Function Neural Networks

Radial Basis Function Neural Network (RBFNN) models use Radial Basis Functions (RBFs) as the activation functions, being composed of one input layer, one hidden layer, and one linear output layer. Park et al. [28] used an RBFNN for short-term freeway traffic volume prediction, with results topping around 64.81% and 91.39%, with forecast traffic volumes being in the 10% and 20% error range, respectively. The prediction accuracy was improved by combining fuzzy C-means with an RBFNN and using a Generalized Regression Neural Network (GRNN) following the work of Park [29], Kuang et al. [30], and Buliali et al. [31]. On top of the GRNN, Buliali et al. used a Leave-One-Out Cross-Validation (LOOCV) method to determine the suitable smoothing factor in order to to avoid overfitting, achieving an RMSE of 16.4. Furthermore, Xiaobin [32] explored the use of Particle Swarm Optimization (PSO) to appropriately select the training parameters of an RBFNN, leading to a significant increase in prediction accuracy with a MAPE of 3.37%. Moreover, both the historical data of the current intersection and adjacent intersections were found to have a significant effect on the performance (Zhu et al. [33]). Table 2 indicates the works found and their primary focus, limitations, and performances.

The performance of an RBFNN depends on the selection of centers and widths. The simplicity of the K-means clustering algorithm, width calculation, and the least mean squares algorithm for weight training make the method faster and efficient (Amin et al. [34]). However, the performance of the RBFNNs depends on the choice of the RBFs’ parameters.

### 3.5. Wavelet Neural Networks

A Wavelet Neural Network (WNN) is essentially an MLFNN model where an additional wavelet function is applied to the hidden layers instead of the traditional sigmoid or tanh activation functions. It takes advantage of the multiscale decomposition of the wavelet transform and the self-learning capability of NNs to represent complex patterns. Ge and Wang [35] proposed a WNN-based short-time traffic flow prediction model that increased the accuracy and facilitated the convergence time, primarily due to the use of small training datasets. To further reduce the running time, Lin et al. [36] employed the use of a KNN to preselect the optimal training datasets for the WNN. Li and Sheng [37] and Yang and Hu [38] placed particular emphasis on the improvement of the prediction accuracy. Li and Sheng proposed a modified adaptive particle swarm optimization algorithm based on cloud theory that exhibited better performance in comparison to other baselines. Yang and Hu combined an Improved Genetic Algorithm (IGA) with a clustering search strategy and a WNN (IGA-WNN), boosting the prediction accuracy and better handling nonlinear cases. Table 3 indicates the works found using WNNs and their focus, limitations, and performances.

Similar to RBFNNs, WNNs require less training effort and the obtained models have a better representation ability than MLFNNs. A significant drawback of the WNN is the limited input dimensions. Constructing a WNN requires a large computational effort in the input decomposition, in particular with the higher dimensionality of the input vector.

### 3.6. Time-Delay Neural Networks

Time-Delay Neural Network (TDNN) models are generally defined as multilayer NNs where the time-shifting approach is used to capture the temporal dynamics of time series data by encoding on delayed inputs or states. Lingras and Mountford [39] and Zhong et al. [40] applied a Genetic Algorithm (GA) in the design of a TDNN for short-term traffic forecasting aimed to handle large coverage areas, obtaining 10% average errors. To improve the accuracy, Wang et al. [41] integrated spatial and temporal autocorrelations of road traffic networks using a Space–time-Delay Neural Network (STDNN) using a low learning rate, achieving a MAPE of 13.7. Khandani and Mikhael [42] included a pretransformed layer with a TDNN using Discrete Cosine Transform (DCT), combined with a mixed transform strategy, to improve the model learning process and increase accuracy significantly. Table 4 summarizes the works found using TDNNs and their primary focus, limitations, and performances.

TDNNs are a simple way to represent correlations between past and present values in a feedforward model, requiring lower computational effort when compared to other models. However, longer a training time and difficulties in capturing temporal dynamics are some of the significant drawbacks of TDNNs.

### 3.7. Recurrent Neural Networks

The Recurrent Neural Network (RNN) models are powerful and robust because of their internal memory and ability to remember the input they receive, which allows them to predict future events. Hence, they are helpful in modeling sequence data such as time series. In the literature, a good amount of works on traffic state prediction were found based on the standard RNN, Long Short-Term Memory (LSTM), and the Gated Recurrent Unit (GRU), which are briefly described in the following sections.

#### 3.7.1. Standard RNNs

Unlike traditional NNs, RNNs are designed by feeding the output from previous steps into the input of the current state cell. They are particularly suitable for predicting future scenarios utilizing the sequential inner characteristics of the data. Ulbricht [43] pioneered the use of RNNs for traffic forecasting, using a multi-recurrent NN, and compared the proposed model with conventional statistical methods. The proposed multi-recurrent NN exhibited improved performance. In order to improve the accuracy, in particular for datasets characterized by instability, dynamic fluctuations, and unpredictability, Yun et al. [44], Dia [45], and Ishak et al. [46] proposed a time-delayed recurrent model, achieving a MAPE of around (4–6)%. Zhang [47] employed autocorrelation and cross-correlation analysis to construct more adequate models, and with careful parameters, optimization improved the overall accuracy. Bohan and Yun [48] applied LSTM, a GRU, and a Bidirectional RNN on the same datasets (GPS data), showing the feasibility of recurrent neural networks to achieve adequate traffic flow forecasting. Table 5 summarizes the works found using RNNs, their focus, limitations, and performances.

One major drawback of the standard RNNs is the exploding and gradient vanishing problems, which cause difficulties in training the models.

#### 3.7.2. Long Short-Term Memory NNs

The Long Short-Term Memory (LSTM) model was proposed to overcome the gradient vanishing problem in traditional RNNs, which prevents the Vanilla RNN from capturing long-term dependencies (Hochreiter et al. [49]). The LSTM model employs a gating mechanism that allows deciding when and how to update its memory state. In the work of Ma et al. [50], an LSTM was applied to automatically determine the optimal time lags and overcome the backpropagation error decay problem. However, they considered only the temporal dependencies to be captured, resulting in relatively high errors and less robustness. Khan et al. [51] addressed incomplete data by utilizing a masking and imputation scheme, achieving a MAPE of 2.10% for annual daily forecasting. Moreover, Jia et al. [52] combined rainfall data in addition to speed data as the input and further improved the robustness and accuracy. Zhao et al. [53] took into consideration the spatiotemporal correlation in traffic using a 2D network, effectively improving both robustness and accuracy. Lu et al. [54] further improved the performance of LSTM by introducing cascading Temporal-aware Convolutional Context (TCC) blocks and a Loss-Switch Mechanism (LSM) to counteract non-Gaussian disturbances effectively. Table 6 summarizes the works found using LSTMs and their limitations and performance.

#### 3.7.3. Gated Recurrent Unit NNs

The Gated Recurrent Unit (GRU), a variation of the LSTM model, was introduced by Cho et al. [55]. Although the performances of LSTM and the GRU are similar in many applications, GRU networks contain fewer parameters and are faster to train. Fu et al. [56] were one of the first to apply a GRU on the PeMS [57] datasets for traffic forecasting, showing slightly better performance and faster convergence than LSTM. To improve the accuracy, Zhao et al. [58] proposed a data fusion method to fuse the information of two different datasets and applied a GRU for travel time prediction. Bartlett et al. [59] considered the computational cost and network structure optimization and proposed three recurrent neural network models, with the GRU model outperforming the others, achieving an RMSE of 9.26%. To further enhance the accuracy and robustness, Pu et al. [60] integrated a decay mechanism as extra gates of the GRU model to handle the missing value problem. Model transferability and reproducibility can be improved by considering both temporal and local features in traffic flow. An attention-based GRU model was proposed by Khodabandelou et al. [61], achieving an MAE of 1.26 for a 1 h data sampling rate. Table 7 indicates works found using GRUs and their focus, limitations, and performances.

### 3.8. Convolutional Neural Networks

A Convolutional Neural Network (CNN) contains layers such as convolution, max pooling, and fully connected layers apart from the input and output layers. The convolution layers in CNNs are connected locally through sliding filters, unlike traditional feedforward NNs, in which one layer is fully connected to the next layer and so on, enabling the extraction of relevant features. Ma et al. [62] proposed a CNN-based network-wide speed prediction model that can convert spatiotemporal traffic dynamics into the image space, outperforming other algorithms with an average accuracy improvement of around 42.91%. Zang et al. [63] further improved the results with the introduction of a three-channel CNN. Although they could slightly improve the training process and accuracy, the robustness was still a concern. In the work of Yu et al. [64], a Spatiotemporal Recurrent Convolutional Network (SRCN) was proposed that explores the advantages of DCNNs and LSTM. To improve the scalability and accuracy, Fouladgar et al. [65] considered a decentralized method where each node can accurately predict in real time based on the neighboring station’s state utilizing a regularized euclidean loss function. Table 8 summarizes the works found using CNNs and their focus, limitations, and performances.

### 3.9. Deep Belief Networks

Deep Belief Networks (DBN) are multiple layers of restricted Boltzmann machines (RBMs) with nondirectional connections between the layers and are able to learn a probability distribution over the input data. Hong et al. [66] proposed a multitask grouping neural network with a regression output layer at the top and a DBN on the bottom that achieved around 91.7% accuracy in traffic flow forecasting. Tan et al. [67] introduced two DBNs, one having Gaussian visible units and hidden binary units and the remaining units being binary, with results showing an improvement in the accuracy, but less robust nonetheless. Chen et al. [68] combined a DBN with Gaussian–Bernoulli restricted Boltzmann machines and a BPNN to improve the accuracy further, but robustness was still a concern. To enhance the prediction accuracy and robustness, Koesdwiady et al. [69] correlated weather parameters and traffic flow by employing a decision-level data fusion scheme. In the work of Bao et al. [4], the weather condition was also used, and the employed Support Vector Regression (SVR) to derive an improved DBN, which showed a good improvement both in robustness and accuracy. Table 9 summarizes the works found using DBNs and their focus, limitations, and performances.

### 3.10. Fuzzy Neural Networks

Fuzzy Neural Networks (FNNs) combine the merits of fuzzy systems and NNs. They can learn membership functions and appropriate fuzzy rules by engaging the adaptive approximation ability of NNs. Additionally, FNN models have better interpretability compared to NN-based models. Yin et al. [70] proposed an online-training-based FNN where the fuzzy approach was used to cluster the data and used an NN to specify the input–output relationships. The results showed good performance, in particular for less traffic fluctuation. Quek et al. [71] introduced a Pseudo-Outer-Product FNN using the Truth-Value-Restriction method (POPFNN-TVR), but it was less capable of counteracting noisy data. Zhao [72] combined an Interval Type-2 Fuzzy Neural Network (IT2FNN) and self-organizing learning algorithm that somehow failed to achieve performance improvement. However, Li [73] was successful in improving the accuracy by introducing Dynamic Fuzzy Neural Networks (D-FNNs) for traffic flow prediction. Still, the model showed a lack of robustness and a relatively slow learning process. Tang et al. [74] mainly aimed at improving the learning ability by suggesting an FNN model with both unsupervised and supervised learning processes, by employing a K-means method and a Gaussian fuzzy membership function; on the other hand, a weighted recursive least squares estimator was used in the supervised learning process. They not only improved the learning ability, but also achieved a 5% improvement in accuracy. In the work of An et al. [75], the focus was given to robustness by proposing a Fuzzy-based Convolutional Neural Network (F-CNN) method to incorporate uncertain traffic accident information, achieving a superior performance compared to other state-of-the-art works. Table 10 summarizes the works found using FNNs with their limitations and performances.

### 3.11. Other NNs

#### 3.11.1. Autoencoders

To solve the problem of the fuzziness and uncertainty of traffic states in a signalized intersection, Stacked Autoencoder (SAE) models are commonly employed. Lv et al. [76] and Yang et al. [77] are two of the pioneers who applied the SAE model to traffic forecasting. They used SAEs to learn generic traffic flow features and trained them in a greedy layerwise fashion. Although the accuracy was promising, the models lacked robustness. Xiang and Chen [78] proposed a denoising SAE model consisting of K-means clustering and deep autoencoder networks to improve the robustness and accuracy, reaching a 91.5% and 88% accuracy in simulation and empirical data, respectively (7.1% better than other decision-tree models). Table 11 indicates the works found using AEs with a focus on their limitations and performance.

#### 3.11.2. Modular Neural Networks

Real-time information can predict link travel times and is suitable for to be employed in Modular Neural Networks (MNNs). Generally, unsupervised clustering techniques and MNNs are used to classify and predict link travel times, respectively. In the work of Park et al. [79], it was found that the MNN could give the best overall results compared to other relevant models. Ishak and Alecsandru [80] proposed multimodal techniques to improve prediction performance, but the results showed a lack of robustness. Vlahogianni et al. [81] suggested an MNN consisting of temporal genetically optimized structures of MLPs and showed a good improvement of accuracy with an MSE of 8.21%. Table 12 indicates the works found using MNNs and their focus, limitations, and performances.

#### 3.11.3. Self-Organizing Neural Networks

These traffic forecasting models are based on Self-Organizing map Neural Networks (SONNs) and Self-Organizing Fuzzy Neural Networks (SOFNNs). Tung and Quek [82] combined the fuzzy approach with a self-organizing neural network and proposed the Generic Self-organizing Fuzzy Neural Network (GenSoFNN) algorithm, which showed encouraging performance, obtaining an MSE of 0.244. Boto-Giralda et al. [83] proposed a SONN model based on a stationary wavelet denoising process and a fuzzy ARTMAP. Ll and Huang [84] proposed a traffic forecasting model using Autoregressive (AR) methods based on a Self-Organizing Map (SOM) neural network, significantly improving the prediction accuracy, yielding considerably better performance than other methods. Table 13 presents the works found using SONNs and their focus, limitations, and performances.

#### 3.11.4. Bayesian Neural Networks

When the Bayesian Combined Predictor (BCP) uses an artificial neural network, it is called a BNN. Such a design intends to combine the strengths of neural networks and stochastic modeling. BNN models can generate a complete posterior distribution and produce probabilistic guarantees of the predictions (Petridis et al. [85]). Chan et al. [86] proposed an Adaptive Particle Swarm Optimization (APSO) utilizing Bayesian regularization to minimize the overfitting problem, showing relevant efficiency improvements in traffic forecasting. To improve the accuracy, Gu et al. [87] proposed an Improved Bayesian Combination Model with Deep Learning (IBCM-DL) to increase not only the accuracy, but also the stability. AlKheder et al. [88] focused on evaluating the impacts of adjacent intersections in terms of the traffic volume and using a BCNN; the authors were able to show improvements in both model coherency and accuracy with an average MSE of 0.003468 during weekdays. Table 14 presents the works found using BNNs and their focus, limitations, and performances.

#### 3.11.5. Resource Allocating Networks

A Resource Allocating Network (RAN) allocates new units and adjusts the parameters of existing units in the learning process. If the network performs poorly on a presented pattern, a new unit is allocated to better represent the pattern, with network parameters being updated only when the results are satisfactory on the presented pattern. Although the RAN is mainly designed for mobile network management, it can also be applied in road traffic forecasting. Chen and Grant-Muller [89] investigated the potential of dynamic neural networks to forecast motorway traffic in normal and abnormal conditions, highlighting the importance of RANs. Bouyahia et al. [90] used the Markov Random Field (MRF) to model and predict the spread of traffic congestion and the Markov Decision Process (MDP) to allocate traffic resources, showing good improvement in accuracy. To further improve the performance, Cui et al. [91] used a RAN in road traffic prediction by employing a controller of the network slice that periodically collected the information to predict future road traffic and applied the Bayesian Attractor Model (BAM) to estimate the required resources. Table 15 presents the works found using RANs and their focus, limitations, and performances.

#### 3.11.6. Generative Adversarial Networks

First introduced by Goodfellow et al. [92], Generative Adversarial Networks (GAN) are composed of two NNs, competing against each other in order to generate new synthetic instances of data that can pass for real data. As a GAN can learn the joint distribution of the data and more effectively address the blurry prediction issue, it can be used to learn the distribution of future traffic flows conditioned on previous traffic flows and the most likely sample from the distribution as the prediction result. Liang et al. [93] proposed a deep Generative Adversarial Architecture (GAA) for network-wide prediction consisting of two LSTMs, and the experimental results showed much better performance compared to a BNN. To further increase the accuracy, Zhang et al. [94] proposed TrafficGAN employing both the CNN and LSTM models, which achieved an MAE of 1.76 during weekdays for a 30 min prediction horizon. In the work of Liang Zhang et al. [95], a Self-Attention Generative Adversarial Network (SATP-GAN) was proposed that used Reinforcement Learning (RL), showing an improvement of 6.5% over baseline methods. Different approaches of integrating rules as inductive biases into deep-learning-based prediction models were evaluated by Li et al. [96], confirming the usefulness of GANs in achieving better performance. Table 16 presents the works found using GANs with their focus, limitations, and performances.

### 3.12. Hybrid Schemes

Hybrid approaches in short-term traffic flow forecasting have been also commonly employed; in fact, most recent works are based on different hybrid approaches due to their higher performances when compared to other methods.

#### 3.12.1. ARIMA, BPNNs, and GARCH

In these approaches, first, the linear features of time series are captured by an ARIMA model. For nonlinear features, a BPNN is then employed. To overcome the BPNN’s disadvantages of slow convergence and to avoid falling into local minima, the Simulated Annealing (SA) algorithm is used (Yang et al. [97]). The joint ARIMA and Generalized Autoregressive Conditional Heteroskedasticity (GARCH) modeling approach can improve short-term ridership forecasting accounting for dynamic volatility, providing not only the expected value, but also, the prediction interval can be obtained (Lin et al. [98], Ding et al. [99]).

#### 3.12.2. KNN-LSTM

Generally, in KNN-LSTM schemes, the KNN is used to capture spatial features and LSTM to model the temporal variability of traffic flow. A two-layer LSTM network can be applied to predict traffic flow, and the final prediction results are obtained by result-level fusion with the rank-exponent weighting method. It exhibits competitive performance when compared with well-known prediction models (Luo et al. [100]). Li et al. [101] introduced a Diffusion Convolutional Recurrent Neural Network (DCRNN), achieving an MAE of 2.07 for a 1 h prediction horizon. Yu et al. [102] proposed a Spatiotemporal Recurrent Convolutional Network (SRCN) combining DCNN and LSTM, which showed superior results both in long- and short-term forecasting. Allström et al. [103] combined both parametric and nonparametric approaches in an ensemble Kalman filter, obtaining a MAPE of 6.1 for a 30 min prediction horizon. Kolidakis et al. [104] combined Singular Spectrum Analysis (SSA) with Artificial Neural Networks (ANNs) to provide proactive decisions to mitigate the economic and environmental impacts of traffic congestion. Table 17 indicates the works found using hybrid schemes and their primary focus, limitations, and performances.

## 4. Traffic Signal Control

**Figure 4:**
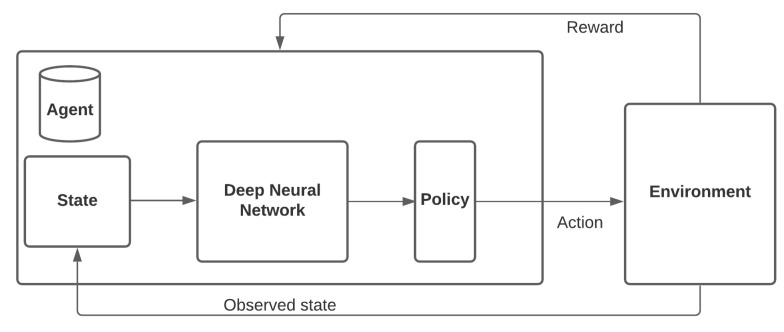
Main blocks of a common deep-reinforcement-learning-based traffic-signal-control model (adapted from Hussain et al. [105]).

In this section, different intersection-traffic-signal-control systems and policies are discussed. In the literature, several strategies and policies were found during our studies, such as fixed-time traffic signal control, i.e., Webster’s method, the SCAT, the SCOOT, Urban Traffic Optimization by Integrated Automation (UTOPIA), ImFlow, MaxPressure, the Generalized Proportional Allocation (GPA), and $P_{0}$. Various machine-learning algorithms and controllers were also identified, such as Q-learning, neural networks, neuro-fuzzy methods, hybrid deep Q-networks, Deep RL, and Boosted GAs. In a multi-agent deep-reinforcement-learning system, traffic light duration is controlled by analyzing independent and shared rewards based on a given objective, for example waiting time and number of waiting vehicles. Figure 4 depicts the main blocks of a common deep-learning-based intersection-traffic-signal-control model.

### 4.1. Fixed-Time Traffic Signal Control

Repeated signal cycles with the same phase structure have been used in fixed-time signal-control methods, which are commonly employed in real-world scenarios, mainly due to their low cost of implementation. By analyzing past traffic data, these methods have their signal parameters calibrated, including phase sequences, cycle lengths, green splits, and offsets (for signal coordination). TRANSYT (Hale [106]) is the most popular of these control methods. Because traffic demand usually varies over time, the Time-Of-Day (TOD) mode is often used, which consists of a collection of distinct signal plans for different times of the day, such as peaks and off-peaks (Zheng et al. [107]). On the other hand, robust signal optimization is used to deal with traffic flow uncertainty, i.e., a scenario-based technique is used in order to ensure the performance of fixed-signal systems (Zhang et al. [108]). For undersaturated and oversaturated demands, the unifying goals of these methods are to minimize vehicle delay and maximize intersection capacity, i.e., vehicle throughput. The signal time now includes queue lengths as well. For example, Jang et al. [109] devised a signal-optimization approach for the equalization of queue growth rates across connections in oversaturated road networks. Osorio and Wang [5] proposed a probabilistic network model to analytically approximate the stationary aggregate joint queue-length distribution of subnetworks. Hence, the developed model could be used to control traffic in cities. Furthermore, spill-backs for signal timings have been considered in recent studies, which have focused on the effects of delay variability. In addition to lowering vehicle delay, signal optimization also aims to minimize delay variability and spill-back likelihood (Mohajerpoor et al. [110]).

#### Webster’s Method

The design of fixed-time (FT) splits under known (historical) constant demand rules by Webster, 1958 [111], and Webster and Cobbe, 1966 [112], has been extensively used in the last 50 years. It is efficient as long as traffic conditions are undersaturated, but fails when queues form in network links due to increasing demand. Kouvelas et al. [113] employed Webster’s procedure within a Traffic-responsive Urban Control (TUC) for real-time operation, and the test implementation showed an average increase of speed by 11.3% compared to Traffic-Actuated Signal plan Selection (TASS) in relatively unsaturated conditions. Aiming at designing traffic signal timing at oversaturated intersections, Eriskin et al. [6] proposed an elimination pairing system and compared the proposal with Webster’s method. The results showed the inefficiency of Webster’s method to handle oversaturated traffic. Ali et al. [114] combined fuzzy logic and the Webster optimum cycle formula, showing an increase of the average waiting time by (18–34)% relative to MaxPressure and fixed-time, respectively. Considering intersection delay, fuel consumption levels, and emissions, Calle-Laguna et al. [115] applied Webster’s method to estimate the optimum cycle length and eventually found an overestimation of the method. Table 18 presents the works found using Webster’s method and their primary focus, limitations, and performances.

### 4.2. Sydney Coordinated Adaptive Traffic System

The Sydney Coordinated Adaptive Traffic System (SCAT) is unique, consisting entirely of computers and being adaptive to traffic demand, with its communication networks providing effective, yet flexible management of the system. The SCAT not only reduces delay, but also improves flow and decreases congestion, leading to a reduction of accidents and petroleum resource use, with the significant benefits of the decrease of air pollution and improving residential amenities (Sims and Dobinson [7]).

### 4.3. Split, Cycle, and Offset Optimization Technique

The Split, Cycle and Offset Optimization Technique (SCOOT) was designed for general applications within computerized urban traffic control systems, responsible for the coordination of the adjustment of the signal timings. An online computer with algorithms calculates and implements the timing predictions from vehicle detectors that are analyzed to minimize congestion. It was found that the SCOOT reduces vehicle delay by an average of 12% when compared to up-to-date optimized FT plans (Hunt et al. [8]). Bretherton [116] and Hansen et al. [117] implemented the SCOOT in different simulation environments to investigate its feasibility for real-time operations. Although the simulation results showed an average delay time reduction by (12–30)%, a performance deterioration was observed with the increased network space. Table 19 presents these works and their focus, limitations, and performances.

### 4.4. Urban Traffic Optimization by Integrated Automation

Urban Traffic Optimization by Integrated Automation (UTOPIA) aims to respond to fluctuations in traffic patterns by adjusting signal timing following traffic demand to reduce traffic congestion, delays, and travel time. Absolute priority assignment is used to select public vehicles and private traffic optimization in all traffic conditions. A deeper analysis demonstrated that the system is capable of handling traffic in heavy traffic conditions, i.e., at peak hours, with gains arising over 35% (Mauro and Di [118]). Wahlstedt [119] and Pavelski et al. [120] simulated UTOPIA using the VISSIM platform to evaluate its potentiality for real-time implementation. The simulation results demonstrated the performance of UTOPIA in the reduction of the average delay time and queue length. Table 20 indicates these works and their focus, limitations, and performances.

### 4.5. ImFlow

ImFlow is a self-optimizing signal system with distributed intelligence and a structure similar to UTOPIA. The optimization is performed in two steps: (i) stage-based optimization at the network/route level based on a cost function with user-defined weights; (ii) signal-group-based optimization at the intersection level based on logical rules; a simulation proved an average reduction of the delay per bus by (26–35)% (Wahlstedt [119]).

### 4.6. MaxPressure/BackPressure Traffic Signal Control

The problems with infrastructure and the cost of centralized approaches motivate the emergence of decentralized control, i.e., a local traffic controller at a given intersection that only requires information from adjacent links; therefore, the required communication infrastructure is minimal. A decentralized algorithm for traffic signal control is MaxPressure (MP), sometimes called BackPressure, which Tassiulas and Ephremides initially developed in 1990 [121], although it was first adopted in urban traffic networks by Varaiya in 2013 [122]. The MP traffic controller, which requires the measurement of queue length, has the advantages of: (i) simple computation; (ii) no need for traffic demand knowledge; (iii) and not requiring the use of a fixed cycle time; instead, time-step-based policies are the actuating method. Le et al. [123] adapted a BackPressure scheme to study its stabilizing efficiency in any traffic demands, and the simulation showed an average reduction of travel time by 20.3%. To increase MP’s accuracy, particularly in high-congestion situations, Gregoire et al. [124] proposed taking into account the queue capacities for the computation of the normalized pressures. Zaidi et al. [125] proposed a multicommodity BackPressure algorithm that showed significant improvement over a Fixed Schedule (FC) controller and a single-commodity backpressure controller in terms of queue length and travel times. Levin and Boyles [126] studied reservation-based intersection-control schemes using MP and $P_{0}$, in particular for autonomous vehicles to improve throughput. Results on the downtown Austin network showed significant performance improvement over other baselines, although they failed to prove it to be actually throughput-optimal. Table 21 indicates these works and their primary focus, limitations, and performances.

### 4.7. Generalized Proportional Allocation Policies

Generalized Proportional Allocation (GPA) policies are decentralized and fully scalable, as they rely on local feedback information only. They do not require any global information about the network topology, the exogenous inflows, or the routing, which makes them robust (Nilsson and Como [127]). Moreover, they consider the overhead time while switching between services (Nilsson and Como [128]). Although GPA is yet to be implemented in real time, Nilsson and Como simulated GPA using the SUMO platform to evaluate its potentiality for real-time operation. The simulation demonstrated a significant improvement in robustness, scalability, and performance relative to other state-of-the-art works. Table 22 presents these works and their focus, limitations, and performances.

### 4.8. $P_{0}$ Policy

The $P_{0}$ policy, first introduced by Smith [131], considers both route costs and stage pressure as a function of flows and green time. When the network is in free-flow conditions, it provides a highly accurate approximation of the maximum throughput. However, under cost imbalance conditions, i.e., when congestion appears, it has some difficulties in approximating the maximum throughput (Cantelmo et al. [132], Smith et al. [133]).

### 4.9. Machine-Learning Approaches for Traffic Signal Control

#### 4.9.1. Q-Learning Controller

Many existing traffic-control systems need a predefined model of the traffic environment to achieve optimal performances. In Q-learning, no prespecified environment model is required, and the relationship among actions, states, and the environment is learned by interaction with the environment. One of the advantages of reinforcement learning is that such algorithms are truly adaptive. They can respond to dynamic sensory inputs from the environment and a dynamically changing environment through ongoing learning and adaptation. Since the one-step Q-learning algorithm updates the Q-estimates at short intervals in conjunction with each action, it is adaptable to inline real-time learning. Furthermore, Q-learning is an off-policy algorithm because it gains valuable experience while exploring actions that may later be nonoptimal. Abdulhai et al. [134] were some of the first to introduce Q-learning in heavily congested intersection traffic signal control, showing encouraging results. Wiring et al. [135] proposed an adaptive optimization algorithm based on RL and compared it against nonadaptive controllers, and better performance was observed mainly for heavy traffic using the Green Light District (GLD) simulator. To maximize throughput, Wunderlich et al. [136] proposed a Longest-Queue-First Maximal-Weight-Matching (LQF-MWM) algorithm utilizing the arbitrary assignment of high priority that outperformed other baselines in high-load conditions. A five-intersection traffic network was studied by Arel et al. [137] using a multi-agent RL approach, where an autonomous intelligent agent governed each intersection, with experimental results demonstrating the advantages of multi-agent-RL-based control over LQF. Another method to enhance the performance was suggested by Prashanth et al. [138], which, by incorporating multiple timescale stochastic approximation in a policy gradient actor–critic algorithm, obtained better performance than standard Q-learning approaches. In the work of Abdoos et al. [139,140], a relatively large network was modeled using multi-agent systems, exploring Q-learning and holonic Q-learning approaches to control signals. Experimental results demonstrated the superior performance of holonic Q-learning in preventing oversaturation, reducing average delay, and increasing throughput. Information sharing among signal controllers was explored by Aziz et al. [141] by proposing an R-Markov Average-Reward-Technique-based RL (RMART) algorithm that not only outperformed in overcrowded conditions, but also significantly reduced emissions. Genders and Razavi [142] used an asynchronous n-step Q-learning algorithm with two NN hidden layers as the agents, showing a reduction of the total mean delay by 40% without compromising throughput. Table 23 presents these works and their focus, limitations, and performances.

The challenge for all Q-Learning Controllers (QLC) is managing a considerable amount of state-action space. Q-learning without enough training examples has difficulties converging to the optimal point. However, Q-learning is a beneficial method since it includes an online-learning scheme to adapt to new situations.

#### 4.9.2. Neural Network Controller

Artificial Neural Network (ANN) models have been widely used in traffic signal control because of their nonlinear mapping, self-adapting, self-organizing, and self-learning capabilities compared to the traditional methods. They are suitable for modeling the nonlinear characteristics of traffic states. To address the changing traffic patterns, Hua and Faghri [143] proposed a multilayer NN-based traffic-signal-control approach for an isolated intersection that paved the way for future research using ANNs. To improve the timing of traffic signals at intersections, Spall and Chin [144] used an ANN that showed approximately 10% improvement in the mean wait time. Saito and Fan [145] focused on finding optimal signal timing by presenting a feasibility testing platform named the Optimal Traffic Signal Control System (OTSCS), applied to the Optimal Traffic Signal Timing Model (OTSTM) based on an ANN, which reduces the time to reach the optimal solution. Kim et al. [146] studied the applicability of ANNs for the cycle-length design of Adaptive Traffic Control Systems (ATCSs), reducing by 8.3% the cycle length in saturated traffic conditions. Intersection traffic signal control solely based on video images instead of conventional traffic parameters, such as delays and queue lengths, was proposed by Jeon et al. [147], achieving a 23% delay time reduction compared with other baselines. To further enhance the performance, Bernas et al. [148] proposed a neuro-evolution strategy, which, compared with other decentralized baselines, showed a superior reduction of delay time. Table 24 summarizes these works, presenting their focus, limitations, and performances.

#### 4.9.3. Hybrid Approaches

- Neuro-Fuzzy-Based Systems

As a neural network can learn and self-adapt, a fuzzy system deals efficiently with the uncertainty and inaccuracies of real systems by using if–then rules, a hybrid approach consisting of both the neural network and fuzzy logic, generally providing excellent results. Mir and Hassan [149] proposed a neuro-fuzzy-based approach where a Fuzzy Logic System (FLS) was used for model training and an NN was used for the calculation of the green light time, proving the potentiality of an efficient traffic signal control. Dong et al. [150] combined an NN and FLS to derive an Adaptive Fuzzy Neural Network (AFNN) algorithm that reduced the delay time by 8.45% with a 24.04% increase in average fuel economy. To further enhance the performance taking into account the traffic conditions on both the current lane and the adjacent lane, Mittal and Chawla [151] proposed a hybrid neuro-fuzzy-based adaptive system that, in comparison with FLS- and FT-based systems, reduced the intersection waiting time by (22.6–46.37)%. Table 25 presents these works with their primary focus, limitations, and performances.

- Deep Reinforcement Learning

The efficiency and accuracy of traffic signal control systems can be enhanced by fusing Deep Learning (DL) and Reinforcement Learning (RL). This type of approach can deal with large amounts of data processing, systematic perception, and expression, which is crucial to the coordinated control of arterial intersections (Chen et al. [152]). Luo et al. [153] combined DL and RL by utilizing the MDP and CNN, which reduced the queue length by 42.5% relative to DQN. Considering knowledge sharing among the agents, Li et al. [154] proposed the Knowledge-Sharing Deep Deterministic Policy Gradient (KS-DDPG) algorithm, which showed significant efficiency in controlling large-scale networks and coping with fluctuations in traffic flow. The inability of DRL algorithms to meet the demands of coordination among the agents inspired Wang et al. [155] to propose a Cooperative Group-Based Multi-agent reinforcement learning-ATSC (CGB-MATSC) framework that demonstrated a significant reduction of average waiting time by 42.08% relative to FT. Kekuda et al. [156] proposed an n-step State, Action, Reward, State, and Action (SARSA) algorithm to increase the implementability in low-cost real-time systems and compared it with LQF; it showed a 5.5% reduction of the average queue length. Table 26 indicates these works and their focus, limitations, and performances.

- Combination of QL, NNs, and FL

Methods combining Q-learning, BP neural networks, and the fuzzy controller have shown promising efficient traffic signal control performance. In such approaches, QL and BPNNs are used to determine the optimal switching time of a particular phase and the fuzzy controller to select the optimal phase sequence (Zhao et al. [157]).

- Hybrid Deep Q-Networks

A hybrid deep Q-network combines both discrete and continuous DRL approaches to control traffic signals and simultaneously decide the proper phase and its associated duration. This type of framework can reduce the average queue length and travel time by a significant amount. Pálos and Huszák [158] investigated and evaluated DQN, double-DQN, dueling DQN, and double-dueling DQN approaches for traffic signal control based on six objective functions, such as waiting time minimization and average speed maximization, outperforming double-dueling DQN in all aspects. Bouktif et al. [9] customized a Parameterized Deep Q-Network (P-DQN) architecture, and the evaluation results using Simulation of Urban Mobility (SUMO) showed that it surpassed other benchmarks, achieving a reduction of the travel time by 5.78%. In the work of Dampage et al. [159], YOLOv3-tiny was retained and combined with OpenCV, and the traffic density was measured, which drives the signaling schemes using a trained DQN. For a multi-intersection scenario, it achieved an increase of the average speed by 18% compared with a static traffic light system. Table 27 summarizes these works concerning their primary focus, limitations, and performances.

- Boosted Genetic Algorithm

Traffic control optimizations combining Machine Learning (ML) and Genetic Algorithms (GA) are also efficient. In the work of Mao et al. [160], the Extreme-Gradient Decision-Tree (XGBT) and Genetic Algorithm (GA) were combined to reduce the total travel time by almost half when used under incident conditions.

## 5. Discussion

A systematic literature search was performed in the Science Direct, Scopus, and Google Scholar databases with the following keywords in various combinations: “intelligent transportation”, “intelligent traffic management and control”, “image processing and deep learning-based intelligent traffic management and control”, “short-term traffic forecasting”, “image processing and deep learning-based short-term traffic forecasting”, “intersection traffic signal control”, “image processing and deep learning-based intersection traffic signal control”. One-hundred forty-four fully fledged research articles were finally selected based on the following inclusion criteria: most relevant, most cited, and most recent. For traffic state forecasting, in terms of performances, the GAN-based methods and also hybrid approaches showed better performance on state-of-the-art datasets, i.e., PeMS (Li et al. [154], Zhang et al. [95]). For intersection signal control, DRL- and DQN-based approaches showed much better efficiency and robustness (Wang et al. [155], Bouktif et al. [9]) relative to other baselines. However, no model is self-sufficient to address all the problems, and hence, plenty of scope for improvement exists. A comparative analysis followed by a very brief summary of the fundamental research challenges is presented in the following sections.

### 5.1. Why Deep Learning?

Traffic states are generally affected by long-term and short-term traffic features. As an example, during the weekdays, traffic flow will always show a rapid increment and decrement in the morning and evening, respectively, referred to as long-term features, because it is affected by society’s behaviors. There might be uncertain fluctuations due to adverse weather, traffic accidents, and other nonrecurrent events, which are called short-term features. For a model to capture these features, a considerable amount of data must be processed efficiently.

Moreover, the corrupted or missing value problem is common in time series data, which is difficult to address by traditional machine-learning approaches. Additionally, the traffic states of the intersections are interrelated with their adjacent counterparts. An efficient intersection traffic signal control demands perceiving of the environments correctly, to take actions accordingly in a coordinated manner. Traditional machine-learning approaches have limitations in handling these demands. Deep-learning-based methods, on the contrary, have a much better ability to overcome these problems efficiently.

### 5.2. Comparative Analysis

**Figure 5:**
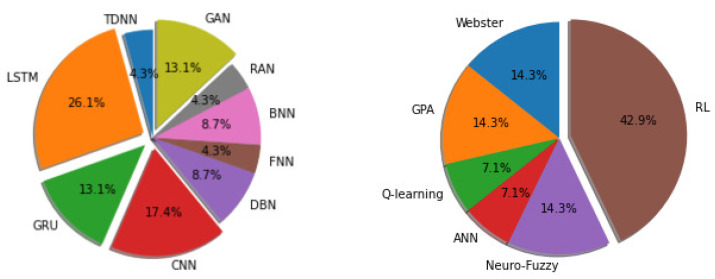
Comparisons among different methods in terms of occurrence between the years 2019 and 2021: the graph on the left is traffic state prediction, and the one on the right is intersection traffic signal control.

One way to find out whether a method in ITMC is efficient or not is to analyze the number of documents published in recent times based on those techniques. In recent years, mainly from 2019, researchers have been applying the LSTM, GRU, CNN, GAN, DBN, FNN, BNN, RAN, and TDNN approaches in traffic state prediction and for intersection signal control. The approaches of RL, GPA, Hybrid, ANN, and Webster’s method have been deployed. For traffic state prediction, out of the 71 studied articles, 39.2% of the works published between 2019 and 2021 were based on RNNs (LSTM and GRU more precisely). On the other hand, for intersection traffic signal control, 42.9% of the works out of 73 utilized reinforcement learning-based methods (DQN and DRL) within the same time horizon, as is depicted in Figure 5.

However, the best way to judge the suitability of a method is to analyze it in terms of its performance. CNN-based traffic state prediction methods trained on datasets without any missing values revealed superior performance compared to other baselines. This is because CNN-based models can capture the spatiotemporal features more efficiently than other models. For example, a SRCN-based forecasting model achieved an RMSE of 4.32 on the PeMSD7 dataset (Yu et al. [64]). One major problem with this kind of method is robustness. With the arrival of nonrecurrent events such as congestion, their performance deteriorates. To achieve higher robustness, LSTM-based methods with an effective mechanism to counteract non-Gaussian disturbances showed much better performance relative to other methods, for example, in the work of Lu et al. [54].

On the contrary, a considerable amount of time series data is required for good performance, and it is challenging to find datasets without any missing value problems. The GAN-based approaches overcome these problems by providing new artificial data of the same quality as the training data. Furthermore, the CNN and LSTM embedded GAN-based methods have shown the best performance so far, by achieving an RMSE of 2.12 for prediction over a long time horizon (Zhang et al. [94]).

Multi-agent deep-reinforcement-learning-based methods are expected to be dominant over other state-of-the-art methods for intersection traffic signal control. Traffic states are highly unpredictable, and the states of an intersection depend on others. Hence, coordination among the different intersections is essential. Multi-agent deep-reinforcement-learning-based methods possess the provision to cope with this. For example, the knowledge-sharing deep deterministic policy gradient algorithm showed an average reduction of the queue length and intersection delay by 28.9% and 35.1%, respectively, relative to the MaxPressure (MP) method (Li et al. [154]).

### 5.3. Research Challenges

#### 5.3.1. Need for Better Datasets

Most of the works studied in the literature used personally collected datasets, as quality fully publicly available datasets are scarce. Nonetheless, PeMS from the Caltrans Performance Measurement System was mainly used by the researchers (Li et al. [101], Lu et al. [54]). The publicly available datasets found during our study are indicated in Table 28.

#### 5.3.2. Reduction of Computational Complexity

Most state-of-the-art models, in particular DL, typically require millions of parameters and billions of operations to produce human-level accuracy. The memory and computational requirements, in particular the deployment of low-power embedded platforms with lower power budgets, are challenging (Maghazeh et al. [161]). Cloud-based infrastructures are a viable solution to this problem. However, privacy implications, the consumption of a significant amount of power, latency, and scalability are significant drawbacks that need to be addressed (Duan [162]).

#### 5.3.3. Model Interpretability

Deep NNs have been found to be very efficient in handling the complex nature of traffic. However, the complexity of the models often makes the understanding of the prediction results difficult, and issues arise about these models’ accuracy. The combination of FLS and NNs provides better model interpretability (Tang et al. [74]). However, with the increase in traffic complexity, they fail to provide optimal outputs. Hence, there are plenty of opportunities to enhance the models’ interpretability.

#### 5.3.4. Finding the Best Evaluation Methodologies

Different algorithms search for different trends and patterns. One algorithm may not be the best suited across all datasets. To find the best solution, it is necessary to evaluate them. Hence, evaluating how well a model generalizes to new and unseen data is very important. During this study, it was found that the F1-score, true positive rate, Mean Absolute Percent Error (MAPE), Mean Absolute Error (MAE), Root-Mean-Squared Error (RMSE), variance score, and $R^{2}$ value are often used as traffic forecasting model performance indicators. However, the Average Displacement Error (ADE), Final Displacement Error (FDE), and Maximum Distance (MaxDist) were also found to be used in some recent works.

**Figure 6:**
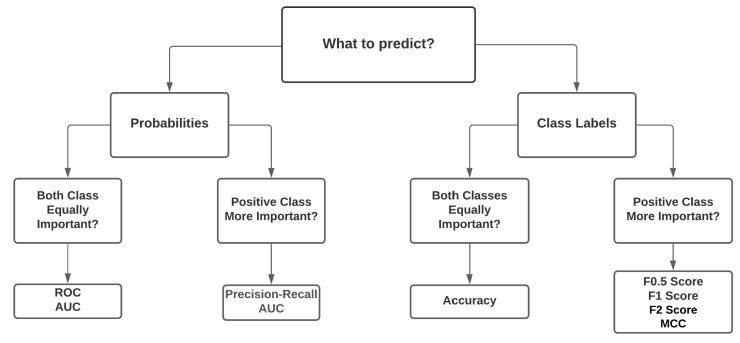
Selection process of the best evaluation metrics for different perspectives.

On the other hand, modern researchers use the average waiting time, queue length, travel time, intersection delay, and fuel economy for intersection traffic signal control. Figure 6 depicts the typically used evaluation metrics in different scenarios based on the studied works. Problems related to probability prediction, the Receiver Operating Characteristic (ROC), and the Area Under the Curve (AOC) are most suitable. While for class labels prediction, evaluation metrics should be selected based on the importance of the classes. For example, if all classes are equally important, “accuracy” can be used as an evaluation metric; otherwise, the F1-score, F2-score, and Matthews Correlation Coefficient (MCC) were found to be convenient. However, which one would be most suited to a particular problem or whether it is necessary to find new evaluation techniques needs to be addressed further.

To evaluate the intersection signal control methods/strategies, powerful simulation environments are utilized by researchers. These simulation tools are helpful for testing and assessing different dynamic transportation issues that are challenging to solve in the real world. On top of that, simulation environments can replace actual experiments with trustworthy representations of the subject matter in a controllable computer program and allow researchers to compare algorithms and reproduce experiments. In most of the recent works, for example, in Nilsson and Como [130], Bouktif et al. [9], and Li et al. [154], the researchers employed the SUMO environment to evaluate their proposed methods. Table 29 indicates the simulation environments found during this study.

#### 5.3.5. Environmental Challenges

GPUs are often used to train and test NNs to deliver the highest arithmetic performance for 32 bit floating-point NN inference. However, operating at 200+ W, their use is becoming prohibitively expensive in terms of energy footprint. Research showed that the carbon footprint of NNs using GPUs is about five-times the lifetime emissions of an average car (Strubell et al. [163]).

## 6. Conclusions

Forecasting traffic and intersection signal control are vitally important for an efficient, ITMC system. For forecasting, the data-driven approaches are gaining popularity because of their higher prediction power and accuracy. However, missing or imbalanced datasets impose difficulties in finding the optimal models. GANs can overcome these difficulties by generating new artificial data that approximate the same unknown distribution as found in the limited training data examples. For intersection signal control, multi-agent deep reinforcement learning and deep Q-networks can be explored in more detail to efficiently control multi-intersection traffic. In summary, with the advancement of image-processing and deep-learning technologies, ITMC research opens a new horizon to enable researchers to address more complex problems in a manageable ways. Therefore, this review aimed to identify the state-of-the-art methods used in ITMC and systematically presented their structure, overall performances, and limitations.

## Figures and Tables

**Table 1:** Summary of the works found using MLFNNs.

Article	Focus	Limitation(s)	Performance(s)
Dougherty and Cobbett, 1997 [23]	Developed a technique of stepwise reduction of the network size by elasticity testing	Occupancy and flow forecasts showed some promise, but not better than naive predictors; speed forecast was much less successful	RMSE: around 0.22 for 15 min flow and occupancy prediction
Florio and Mussone, 1996 [22]	Employed with up to three hidden layers and various input parameters	Filtered data, major amplitude errors in density and flow forecasting	For speed (RMSE: 0.032, MAE: 0.024); for density (RMSE: 0.012, MAE: 0.007); for flow (RMSE: 0.032, MAE: 0.024)
Gilmore and Abe, 1995 [21]	Employed with two hidden layers, trained by a backpropagation technique	Superior forecasting accuracy only for the next 5 min, large training and simulation time	Average accuracy: 85%
Smith and Demetsky, 1994 [20]	Built and tested using real data	Model was not portable, underperformed compared to KNN	Average percentage errors: 7–12)%
Sun et al., 2012 [25]	Several three-layer NNs with different structures, employed the Graphical Lasso (GL) technique	Relatively lower estimation accuracy	RMSE: around 50–170, MARE: around 7–26)% for 31 road links
Kumar et al., 2013 [26]	Increasing the ability of modeling traffic states in heterogeneous traffic	Datasets presented for uninterrupted traffic only; weather conditions, seasonal variation, and extreme conditions were not considered	For 5 min (MAE: 0.628, RMSE: 0.8586, SD: 0.857, r: 0.998); for 15 min (MAE: 1.28, RMSE: 2.92, SD: 2.87, r: 0.998)
Huang et al., 2013 [3]	Data first processed by a clustering method, introduced a correction mechanism	Relatively high MSE	MSE: around (9–10)%
Vlahogianni et al., 2005 [27]	Introduced GA to optimize the structure and to learn the parameters	Less robust	MAE: 6–14, MRPE: 8–22, r: 0.74–0.95
Polson and Sokolov, 2017 [24]	Introduced a deep NN more capable of capturing the nonlinearities	Model interpretability	MSE: 7.7–8.0, $R^{2}$: 0.83–0.85
Elhenawy and Rakha, 2017 [2]	Introduced the discriminative pretraining mechanism	Not fully network-wide	MAPEs of 2.8% and 8% in predicting speed and flow, respectively

**Table 2:** Summary of the works found using RBFNNs.

Article	Focus	Limitation(s)	Performance(s)
Amin et al., 1998 [34]	Centers set using K-means clustering, widths based on the distance from the training data to centers, and weights using the least mean squares algorithm	Required more stability, validation, and verification	Good prediction accuracy with less training time
Park et al., 1998 [28]	Centers were chosen arbitrarily; widths were fixed to a specific value; sped up the learning process	Results were discouraging for real-time implementation	MAPE: (8.82 and 11.9)% (for 2 sites)
Park, 2002 [29]	Employed K-means clustering, fuzzy clustering, and self-organizing maps	Tested with normal traffic volume cases	MAPE: 7.23%, Variance of APE: 38.53
Xiaobin, 2009 [32]	Introduced Particle Swarm Optimization (PSO)	Prediction was not network-wide	MAPE: 3.37%
Kuang et al., 2010 [30]	Used a Generalized Regression Neural Network (GRNN) to find the minimum prediction error	Small datasets	MRE: 7.61%
Zhu et al., 2014 [33]	Input data consisted of the historical data of the current intersection and also adjacent intersections	Prediction was not network-wide	MAPE: 0.13–0.18, MAD: 13.2–15.1, RMSE: 16.4–17.6
Buliali et al., 2016 [31]	Leave-One-Out Cross-Validation (LOOCV) to find the smoothing factor	Prediction was not network-wide	Average MAPE: 3.88%

**Table 3:** Summary of the works found using WNNs.

Article	Focus	Limitation(s)	Performance(s)
Ge and Wang, 2011 [35]	WNN for the improvement of the prediction accuracy and convergence speed	Dataset size: 21 only	Average error of 0.0092
Lin et al., 2013 [36]	Proposed a KNN to form a training set as a preprocessing step	Relatively lower performance	MAPE: 10.70%
Li and Sheng, 2015 [37]	Adopted an adaptive PSO algorithm	Dataset size: 460 only	Prediction error: 0.245
Yang and Hu, 2016 [38]	Combined an Improved Genetic Algorithm (IGA) and WNN (IGA-WNN)	Small datasets	MAE: 11.986, MRE: 0.212

**Table 4:** Summary of the works found using TDNNs.

Article	Focus	Limitation(s)	Performance(s)
Lingras and Mountford, 2001 [39]	Modeled a TDNN using a GA	Higher errors for a low volume of traffic	Average errors: (3–4)%
Zhong et al., 2005 [40]	Modeled a TDNN using a GA and compared with weighted regression models	Less accurate compared to regression models	Average errors: less than 10%
Wang et al., 2016 [41]	Applied a Space–Time-Delay Neural Network (STDNN) to capture the autocorrelation locally and dynamically	Trade-off between model interpretability and training data	MAPE: 0.0401 and $R^{2}$: 0.9734
Khandani and Mikhael, 2019 [42]	Applied a shallow TDNN with pretransformed layers	Higher training time	5% improvement in accuracy

**Table 5:** Summary of the works found using RNN.

Article	Focus	Limitation(s)	Performance(s)
Ulbricht, 1994 [43]	Suggested various types of feedback connections	Low accuracy	RMSE: around 107–152
Yun et al., 1998 [44]	Proposed an Elman network incorporating the time delay in the input layer	Unexpected events were not considered, small dataset	MAPE: 4.4% and 5.8% for highway and urban respectively
Zhang, 2000 [47]	Applied a Jordan network to predict traffic	Small datasets, did not consider unpredictable events	MSE: around 3%
Dia, 2001 [45]	Used PCA	Small datasets, higher error for more than 5 min predictions	MSE: (6–16)%
Ishak et al., 2003 [46]	Implemented and tested an Elman network, a partial RNN, and a TDNN	Lacked robustness	RMSE: around (8–20)%
Bohan and Yun, 2019 [48]	Applied a BRNN, LSTM, and GRU for prediction	Less and low-quality data	MAE of BRNN: around 3%, RMSE of BRNN: around 5%

**Table 6:** Summary of the works found using LSTM.

Article	Focus	Limitation(s)	Performance(s)
Ma et al., 2015 [50]	Applied a three-layer LSTM, flexible and effective capturing of temporal dependency	Did not consider both spatial and temporal information	MAPE: 4.07%, MSE: 5.94 considering both speed and volume data as the input
Jia, et al., 2017 [52]	Incorporated rainfall information	Only temporal information was considered	MAPE: 5.89%
Zhao et al., 2017 [53]	Considered spatial correlations in addition to temporal correlation, confirmed robustness in capturing longer time dependency	Designed only for traffic volume prediction	MRE: around (6–18)% up to 60 min of prediction
Khan et al., 2019 [51]	Applied an RNN, GRU, and LSTM for prediction, masking, and imputation for missing value issues	Lower accuracy for hourly prediction	RMSE of LSTM: 274, 824 and MAPE of LSTM: 18.91%, 2.10% for hourly and annual daily, respectively
Lu et al., 2021 [54]	Captured more distinguishable temporal features and effectively counteracted noise	Applied for isolated points only	RMSE: 21.71–45.25 MAPE: (7.45–12.09)% on the TDAD dataset; RMSE: 16.62–44.40 MAPE: (6.56–12.93)% on the PeMS dataset

**Table 7:** Summary of the works found using GRU.

Article	Focus	Limitation(s)	Performance(s)
Fu et al., 2016 [56]	Created a unique model for each of the traffic flow series	Relatively low accuracy	MAE: 17.21, MSE: 668.9
Zhao et al., 2018 [58]	Showed that a GRU can achieve better accuracy with data fusion	Higher prediction error during rainy days	MAPE: 4.0%, RMSE: 30.01
Bartlett et al., 2019 [59]	Applied an RNN, LSTM, and GRU on a real dataset, developed STATS	Trade-off between accuracy and speed	RMSE of GRU: 9.26%
Pu et al., 2020 [60]	Integrated a decay mechanism to GRU, mitigated negative impact of wet or icy road conditions	Relatively low accuracy and interpretability	MSE: 0.0102, MAE: 0.0783, MAPE: 14.61% with 20% missing data
Khodabandelou et al., 2021 [61]	Proposed an attention-based GRU considering model transferability and reproducibility	Able to predict only speed; prediction was not network-wide	MAE: 1.26, MAPE: 3.0%, RMSE: 1.41 for a 1 h sampling rate

**Table 8:** Summary of the works found using CNNs.

Article	Focus	Limitation(s)	Performance(s)
Ma et al., 2017 [62]	Transformed network to gray-scale images and employed a CNN to predict traffic speed	Relatively slow training rate	MSE: 22.8–38.8 depending on the datasets
Zang et al., 2017 [63]	Employed different channels, including red, green, and blue for color images	Relatively less robust, low training efficiency	MAPE: 0.1768, RMSE: 6.4905 for long-term prediction
Yu et al., 2017 [64]	Able to capture both temporal and spatial features	Higher error for a relatively long time prediction	MAE: 2.37–3.97, MAPE: (5.56–9.73)%, RMSE: 4.32–7.45 on PeMSD7
Fouladgar et al., 2017 [65]	Proposed a decentralized method, introduced a regularized euclidean loss function	Relatively high prediction error in the case of congestion	RMSE: (4–5)%

**Table 9:** Summary of the works found using DBNs.

Article	Focus	Limitation(s)	Performance(s)
Hong et al., 2014 [66]	Proposed a DBN at the bottom and a multitask regression output layer at the top	Prediction was not network-wide	Achieved nearly 5% improvements with an accuracy of 91.7%
Koesdwiady et al., 2016 [69]	Employed weather information	Less robust	Average MAE: 0.0405 and RMSE: 0.0603
Tan et al., 2016 [67]	Used a DBN with Gaussian visible and Binary hidden units (G-B DBN)	Poor performances in evening peak hours	MAPE: 8.48%, RMSE: 6.3 for 30 min prediction time
Chen et al., 2020 [68]	Used Gaussian–Bernoulli restricted Boltzmann machines to build a DBN	Considered data from a single route, less robust	MAE: around (2–12)% during off-peak and peak hours
Bao et al., 2021 [4]	Improved robustness by incorporating weather conditions	Relatively high computation time	MAPE: around 9%

**Table 10:** Summary of the works found using FNNs.

Article	Focus	Limitation(s)	Performance(s)
Yin et al., 2002 [70]	Modeled with a gate network using a fuzzy-set-based approach and an expert network using an NN, adopted an online training method	Performance dropped in the case of traffic state fluctuation	Average RMSE: 2.73
Quek et al., 2006 [71]	Used a Truth-Value-Restriction method (POPFNN-TVR)	Networks became unstable and predicted outputs unreliable with the increase of the noise level	$R^{2}$ Score: around (0.60–0.84)%
Zhao, 2012 [72]	Modeled using interval type-2 fuzzy sets	Prediction was not network-wide	RMSE of occupy rate: 0.138–0.178
Li, 2016 [73]	Used a five-layered dynamic FNN; the network structure was generated during the training process	Prediction error was higher under suboptimal conditions	Normalized RMSE: 0.2683
Tang et al., 2017 [74]	Suggested an FNN based on the Takagi–Sugeno fuzzy inference system	Did not consider nonrecurrent events	MAE: 2.47–3.72, MAPE: (5.3–8.8)%, RMSE: 3.37–5.4
An et al., 2019 [75]	Applied an F-CNN with uncertain traffic accident information	Relatively less prediction accuracy during congestion	MAE: 9.96, MSE: 293.91, RMSE: 17.14

**Table 11:** Summary of the works found using AEs.

Article	Focus	Limitation(s)	Performance(s)
Lv et al., 2014 [76]	Developed an SAE model with up to four hidden layers	Di not considered nonrecurrent events	MAE: 122.8, MRE: 6.21%, RMSE: 183.9
Yang et al., 2016 [77]	Taguchi method for structure optimization and Levenberg–Marquardt algorithm for fine-tuning	Low performance for data with a highly smooth distribution	MAPE: 0.092–0.211

**Table 12:** Summary of the works found using MNNs.

Article	Focus	Limitation(s)	Performance(s)
Ishak and Alecsandru, 2004 [80]	Proposed a model with different MLFNN modules acting as local expert networks and a gating network for combing the results	Relatively small datasets, less robust	Highest reduction of the RMSE: 14 mph, average improvement of the AARE: (6–8.2)%
Vlahogianni et al., 2007 [81]	Proposed temporal genetically optimized structures of the MLP	Significantly time-consuming training	MSE: 8.21%

**Table 13:** Summary of the works found using SONNs.

Article	Focus	Limitation(s)	Performance(s)
Tung and Quek, 2002 [82]	Proposed the GenSoFNN with built-in mechanisms to identify and prune redundant and obsolete rules	The accuracy of the predictions decreased as the time interval increased	Normalized MSE: 0.244
Boto-Giralda et al., 2010 [83]	Based on ARTMAP, capable of performing unsupervised learning tasks	Relatively less training datasets	Average MAPE: 6.9 and RMSE: 58.43%

**Table 14:** Summary of the works found using BNNs.

Article	Focus	Limitation(s)	Performance(s)
Chan et al., 2012 [86]	Bayesian regularization to minimize overfitting, data preprocessed using an exponential smoothing technique, model trained by the Levenberg–Marquardt algorithm	Relatively small datasets	MAE: around 15–25, RMSE: around 16–30
Gu et al., 2019 [87]	Proposed an Improved Bayesian Combination Model with Deep Learning (IBCM-DL)	Did not consider nonrecurrent events	MAE: 7.41, MAPE: 12.44%, VAPE: 13.94%
AlKheder et al., 2021 [88]	Provided a coherent and accurate model for adjacent intersections	Lower performance during weekends	Average MSE: 0.003468, Regression (R): 0.98113 during weekdays

**Table 15:** Summary of the works found using RANs.

Article	Focus	Limitation(s)	Performance(s)
Chen and Grant-Muller, 2001 [89]	Gaussian basis as activation functions, adopted the sequential learning method	Small datasets, less robust	PAE: approximately 9.5%
Bouyahia et al., 2019 [90]	Used MDP to automatically allocate traffic resources	Relatively less robust	Algorithm accuracy, *r*: (82.3–88.5)% depending on the population size

**Table 16:** Summary of the works found using GANs.

Article	Focus	Limitation(s)	Performance(s)
Liang et al., 2018 [93]	One LSTM acted as the generative network and the other as the discriminative network; two NNs trained simultaneously using BP	Traffic-flow phenomena such as breakdown and capacity drop cannot be caught	MAPE: 4.68% and 5.95% for density and flow prediction, respectively
Zhang et al., 2021 [94]	Proposed a CNN and LSTM embedded network-scale deep-traffic-prediction model (TrafficGAN)	Designed for one-step prediction only	MAE: 1.76, MRE: 9.72%, RMSE: 2.12% during weekdays (30 min prediction)
Liang Zhang et al., 2021 [95]	Proposed a Self-Attention Generative Adversarial Network (SATP-GAN)	Considered one intersection only	MAE: around 20, RMSE: around 30 (6.5% improvement)
Li et al., 2021 [96]	Proposed a novel approach for integrating rules as inductive biases into DL models	Did not consider rule priorities and trajectory uncertainty	Mean ADE (meters): 2.53, mean FDE (meters): 5.74, mean MaxDist (meters): 5.99

**Table 17:** Summary of the reference works on hybrid schemes.

Article	Focus	Limitation(s)	Performance(s)
Allström et al., 2016 [103]	Combined parametric and nonparametric prediction techniques in an ensemble Kalman filter	Relatively less robust; prediction was not network-wide	MAPE: 5.7 and 6.1 for 15 and 30 min horizon, respectively
Li et al., 2017 [101]	Introduced the DCRNN, captured spatiotemporal dependency using bidirectional random walks and an encoder–decoder architecture	Did not consider an evolving graph structure	For 1 h prediction MAE: 2.07, RMSE: 4.74, MAPE: 4.9% on PEMS-BAY
Yu et al., 2017 [102]	Proposed the SRCN combining the DCNN and LSTM	Relatively less robust, did not consider nonrecurrent events	MAPE: 12.7
Kolidakis et al., 2019 [104]	Combined Singular Spectrum Analysis (SSA) with the ANN	Relatively less robust, data preprocessing not explored	RMSE: 0.2642, MAE: 0.1752, $R^{2}$: 0.9645

**Table 18:** Summary of the works found based on the Webster’s method.

Article	Focus	Limitation(s)	Performance(s)
Kouvelas et al., 2011 [113]	Simulated Traffic-responsive Urban Control (TUC) strategies with Webster’s method	Good performance for undersaturated conditions only	Increased the mean speed by 11.3%
Eriskin et al., 2017 [6]	EPS used for solving the green times in oversaturated intersections	Field tests were not performed	75.6% improvement of optimum cycle length
Ali et al., 2020 [114]	Combined fuzzy logic and Webster’s optimum cycle formula	Field tests were not performed	Performance enhancement in the average waiting time by about (18–34)%
Calle-Laguna et al., 2019 [115]	Proposed a new model in determining optimum cycle lengths considering vehicle fuel consumption and emission levels	Weaker explanatory models for fuel consumption	Model prediction power for delays ($R^{2}$): 0.78, difference between the delay and fuel-optimum cycle lengths: 11%

**Table 19:** Works found on the SCOOT.

Article	Focus	Limitation(s)	Performance(s)
Bretherton, 1990 [116]	Tested and evaluated using the floating car survey technique	Performance deteriorated when signals were widely spaced and flows were low	Reduction of delay by 12%
Hansen et al., 2000 [117]	Implemented in the CORSIM Simulation Environment	Considered only one small network	Reduction in delay and number of stops by (20–30)%

**Table 20:** Some reference works using UTOPIA.

Article	Focus	Limitation(s)	Performance(s)
Wahlstedt, 2013 [119]	Implemented and tested in VISSIM simulator	Field tests were not performed; extra delay for congested conditions	Average delay per bus was reduced by (14–21)%
Pavleski et al., 2017 [120]	Implemented and evaluated in the VISSIM simulator	Not compared with other baselines	Delay: 67 s, $Q_{Len}$: 55.4 m for traffic flow (veh/h) of 5110

**Table 21:** Summary of the works found related to MP.

Article	Focus	Limitation(s)	Performance(s)
Le et al., 2015 [123]	Adopted a fixed-cycle-time policy with provable stability	Avoided nonconstant switching times, finite link travel time, and link capacity	Average travel time reduction by 20.3%
Gregoire et al., 2014 [124]	Proposed a solution to the nonwork conservation and congestion propagation situations of MP	Performance deterioration for very high arrival rates	Max. $Q_{Len}$: around 2500 s (28.5% reduction than FC)
Zaidi et al., 2016 [125]	Proposed multicommodity and adaptive routing algorithms	Implementation requires communication from every vehicle to the traffic controller	Superior performance in terms of travel time, queue length, and trips completed
Levin and Boyles, 2017 [126]	Adapted pressure-based policies for reservations in dynamic traffic assignments	Not throughput-optimal	Average travel time (veh/min): 7.46 when demand was 100%

**Table 22:** Summary of the works found related to GPA.

Article	Focus	Limitation(s)	Performance(s)
Nilsson and Como, 2019 [129]	Validation and performance evaluation using SUMO	Less robust	Around 7% reduction of total travel time
Nilsson and Como, 2020 [130]	Improvement of the robustness of GPA	Relatively less stable	Improved robustness

**Table 23:** Summary of the works found related to the Q-learning controller.

Article	Focus	Limitation(s)	Performance(s)
Wiering et al., 2004 [135]	Focused on minimizing average traveling, or waiting, times using RL	Implementation suffers from saturation and oscillation	Around 25% reduction in waiting time
Abdulhai et al., 2003 [134]	Introduced Q-learning to find the optimal control of heavily congested traffic	Considered only one isolated two-phase signal	Reduction of delays by (38–44)%
Wunderlich et al., 2008 [136]	Utilized a Maximal Weight Matching algorithm (LQF-MWM) to minimize the queue sizes	Reduction of delays to a entity resulted in a significant increase in delays to others	Outperformed other models, in particular at high traffic loads
Arel et al., 2010 [137]	Compared MARL with LQF in isolated and multi-intersection networks	Considered smaller traffic networks	Reduction of average delay (veh/s): around 21.9% for a high arrival rate
Prashanth et al., 2011 [138]	Proposed a Policy Gradient Actor–Critic algorithm (PG-AC-TLC)	Implementation problem on larger road networks	Around 40% reduction in average junction waiting time
Abdoos et al., 2011 [139], 2013 [140]	Modeled relatively large and nonregular traffic network based on Q-learning	Action space with relatively fewer parameters	Reduction of average delay by (11.7–43.7)%
Aziz et al., 2018 [141]	Applied RMART leveraging information sharing among signal controllers	Algorithm complexity and evaluation	Queue length reduction: 36%, average delay reduction: 22% for high congestion
Genders and Razavi, 2019 [142]	Applied RL with function approximation to train, used asynchronous n-step Q-learning algorithm for the agent	Used a very simple function approximator, yielding lower delays for vehicles in left-turn lanes	Reduced mean total delay by up 40% without compromising throughput

**Table 24:** Summary of the works found using ANNs.

Article	Focus	Limitation(s)	Performance(s)
Hua and Faghri, 1995 [143]	Proposed a multilayered NN architecture for traffic signal control	Missing an evaluation, as well as a comparison with other baselines	For 800 VPH: reduction of average delay and number of stops by 65.25% and 45.8%, respectively
Spall and Chin, 1997 [144]	Presented a System-wide Traffic Adaptive Control (S-TRAC) method based on an ANN	Simulated with less volume of traffic, missing comparison with other baselines	Approximately 10% improvement of mean wait time
Saito and Fan, 2000 [145]	Developed the OTSCS software, combined heuristic optimal signal timing with ANN	Considered only two-phase signalized intersections, less training data	Reduction of optimal delays by 49.65%
Kim et al., 2008 [146]	Proposed an ANN model able to deal with various saturation levels	Relatively low and simulated datasets	Around 8.3% reduction of cycle length for traffic over 6000 VPH
Jeon et al., 2018 [147]	Used only video images of an intersection to represent its traffic state	Applicable to a single independent intersection only	Average delay was reduced by more than 23%
Bernas et al., 2019 [148]	Proposed a decentralized system that evaluated priorities	Less efficient vehicle location detection approach	Average delay reduced by (4–40)% compared with other baselines

**Table 25:** Summary of the works found combining NNs and FLS.

Article	Focus	Limitation(s)	Performance(s)
Mir and Hassan, 2018 [149]	Combined FLS and an NN where FLS was used for training and the NN for green light control	Less training datasets	Demonstrated the potentiality of the approach in signal control
Dong et al., 2019 [150]	Proposed an AFNN where waiting vehicles were detected using Vehicle to X (V2X)	Relatively small training datasets	Average delay time reduced by 8.45% and fuel economy increased by 24.04%
Mittal and Chawla, 2020 [151]	Combined an NN and FLS considering traffic conditions on the current and adjacent lane	Applicable to a single independent intersection only	Reduction of average waiting time by (22.6–46.37)%

**Table 26:** Summary of the works found using DRL.

Article	Focus	Limitation(s)	Performance(s)
Luo et al., 2020 [153]	Modeled as a Markov Decision Process (MDP); a CNN was used to map the states to the rewards	Converged slightly more slowly	Reduction of the queue length by 42.5% compared to the DQN
Li et al., 2021 [154]	Proposed the knowledge-sharing deep deterministic policy gradient algorithm	Limited overall communication efficiency	Average reduction of the queue length by 28.9%, intersection delay by 35.1%, and number of stops by 21.0% compared with the MP
Wang et al., 2021 [155]	Introduced Cooperative Group-Based Multi-agent reinforcement learning-ATSC (CGB-MATSC)	Less scalable with high training costs	Reduction of the average waiting time by 42.08% relative to FT
Kekuda et al., 2021 [156]	Proposed a low-cost real-time system using an n-step SARSA algorithm	Considered a risk-insensitive approach	Reduction of the queue length by 5.5% relative to LQF

**Table 27:** Summary of the works found using hybrid DQNs.

Article	Focus	Limitation(s)	Performance(s)
Pálos and Huszák, 2020 [158]	Examined the DQN, double-DQN, dueling DQN, and double-dueling DQN	Considered a single intersection environment only	Reduction of the waiting time by 34.2% and 5.11% relative to the DQN and double-DQN, respectively
Dampage et al., 2020 [159]	Proposed a retrainedYOLOv3-tiny, OpenCV, and DQN-based coordinated system, signaling schemes based on traffic density	Not robust; performance deteriorated with high congestion	For multi-intersection scenarios, 18% increase of the average speed compared to a static traffic light system
Bouktif et al., 2021 [9]	Customized a Parameterized Deep Q-Network (P-DQN) architecture	Considered a single intersection environment only	Reduced the average queue length and travel time by 22.20% and 5.78%, respectively

**Table 28:** List of the publicly available datasets found during this study.

Name	Description
Caltrans Performance Measurement System (PeMS) https://pems.dot.ca.gov/ (accessed on 16 November 2021)	Collected in real time from over 39,000 individual detectors in California and providing over 10 years of data for historical analysis
Open Data (VDOT) https://www.virginiaroads.org/datasets/VDOT::traffic-volume/about (accessed on 16 November 2021)	Consists of ADT and AAWDT volumes with vehicle classification data for most recent years from the Virginia Department of Transportation
(pNEUMA) https://open-traffic.epfl.ch/ (accessed on 16 November 2021)	Urban datasets of naturalistic trajectories of half a million vehicles in the downtown area of Athens, Greece
(IDOT) http://www.travelmidwest.com/ (accessed on 16 November 2021)	Traffic flow data from the Illinois Department of Transportation containing averaged speed, flow, and occupancy
(TDAD) http://www.its.washington.edu/tdad/ (accessed on 16 November 2021)	Traffic Data Acquisition and Distribution system of the Washington State Department of Transportation

**Table 29:** Simulation environments found during this study.

Name	Description
(SUMO) https://sumo.dlr.de/docs/index.html (accessed on 16 November 2021)	Simulation of Urban Mobility
(PTV VISSIM) https://www.ptvgroup.com/en/solutions/products/ptv-vissim/ (accessed on 16 November 2021)	Planung Transport Verkehr Vision Traffic Suite
(Aimsun) https://www.aimsun.com/ (accessed on 16 November 2021)	Advanced Interactive Microscopic Simulator for Urban and Non-Urban Networks
(MATSim) https://www.matsim.org/ (accessed on 16 November 2021)	Multi-Agent Transport Simulation
(TRANSIMS) https://code.google.com/archive/p/transims/ (accessed on 16 November 2021)	Transportation Analysis and Simulation System

## Data Availability

Not applicable.

## References

[1] Chen S., Kuhn M., Prettner K., Bloom D.E. (2019). The global macroeconomic burden of road injuries: Estimates and projections for 166 countries. Lancet Planet. Health.

[2] Elhenawy M., Rakha H. (2017). Spatiotemporal traffic state prediction based on discriminatively pre-trained deep neural networks. Adv. Sci. Technol. Eng. Syst..

[3] Huang H., Tang Q., Liu Z. (2013). Adaptive correction forecasting approach for urban traffic flow based on fuzzy-mean clustering and advanced neural network. J. Appl. Math..

[4] Bao X., Jiang D., Yang X., Wang H. (2021). An improved deep belief network for traffic prediction considering weather factors. Alex. Eng. J..

[5] Osorio C., Wang C. (2017). On the analytical approximation of joint aggregate queue-length distributions for traffic networks: A stationary finite capacity Markovian network approach. Transp. Res. Part B Methodol..

[6] Eriskin E., Karahancer S., Terzi S., Saltan M. (2017). Optimization of traffic signal timing at oversaturated intersections using elimination pairing system. Procedia Eng..

[7] Sims A., Dobinson K. SCAT the Sydney coordinated adaptive traffic system: Philosophy and benefits. Proceedings of the International Symposium on Traffic Control Systems.

[8] Hunt P., Robertson D., Bretherton R., Winton R. (1981). SCOOT-a Traffic Responsive Method of Coordinating Signals.

[9] Bouktif S., Cheniki A., Ouni A. (2021). Traffic signal control using hybrid action space deep reinforcement learning. Sensors.

[10] Nagy A., Simon V. (2018). Survey on traffic prediction in smart cities. Pervasive Mob. Comput..

[11] Pasquale C., Sacone S., Siri S., Ferrara A. (2019). Traffic control for freeway networks with sustainability-related objectives: Review and future challenges. Annu. Rev. Control.

[12] Mirchandani P., Wang F.Y. (2005). RHODES to intelligent transportation systems. IEEE Intell. Syst..

[13] Do L.N., Taherifar N., Vu H.L. (2019). Survey of neural network-based models for short-term traffic state prediction. Wiley Interdiscip. Rev. Data Min. Knowl. Discov..

[14] Box G.E., Jenkins G.M., Reinsel G.C., Ljung G.M. (2015). Time Series Analysis: Forecasting and Control.

[15] Irhami E.A., Farizal F. (2021). Forecasting the Number of Vehicles in Indonesia Using Auto Regressive Integrative Moving Average (ARIMA) Method.

[16] Li C., Xu P. (2021). Application on traffic flow prediction of machine learning in intelligent transportation. Neural Comput. Appl..

[17] Mingheng Z., Yaobao Z., Ganglong H., Gang C. (2013). Accurate multisteps traffic flow prediction based on SVM. Math. Probl. Eng..

[18] Yu B., Wang Y., Yao J., Wang J. (2016). A comparison of the performance of ANN and SVM for the prediction of traffic accident duration. Neural Netw. World.

[19] Yu H., Ji N., Ren Y., Yang C. (2019). A special event-based K-nearest neighbor model for short-term traffic state prediction. IEEE Access.

[20] Smith B.L., Demetsky M.J. Short-term traffic flow prediction models-a comparison of neural network and nonparametric regression approaches. Proceedings of the IEEE International Conference on Systems, Man and Cybernetics.

[21] Gilmore J.F., Abe N. (1995). Neural network models for traffic control and congestion prediction. J. Intell. Transp. Syst..

[22] Florio L., Mussone L. (1996). Neural-network models for classification and forecasting of freeway traffic flow stability. Control Eng. Pract..

[23] Dougherty M.S., Cobbett M.R. (1997). Short-term inter-urban traffic forecasts using neural networks. Int. J. Forecast..

[24] Polson N.G., Sokolov V.O. (2017). Deep learning for short-term traffic flow prediction. Transp. Res. Part C Emerg. Technol..

[25] Sun S., Huang R., Gao Y. (2012). Network-scale traffic modeling and forecasting with graphical lasso and neural networks. J. Transp. Eng..

[26] Kumar K., Parida M., Katiyar V. (2013). Short term traffic flow prediction for a non urban highway using artificial neural network. Procedia Soc. Behav. Sci..

[27] Vlahogianni E.I., Karlaftis M.G., Golias J.C. (2005). Optimized and meta-optimized neural networks for short-term traffic flow prediction: A genetic approach. Transp. Res. Part C Emerg. Technol..

[28] Park B., Messer C., Urbanik I. (1998). Short-Term Freeway Traffic Volume Forecasting Using Radial Basis Function Neural Network. Transportation Research Record 1651 TRB.

[29] Park B.B. (2002). Hybrid neuro-fuzzy application in short-term freeway traffic volume forecasting. Transp. Res. Rec..

[30] Kuang X., Xu L., Huang Y., Liu F. Real-time forecasting for short-term traffic flow based on general regression neural network. Proceedings of the 2010 8th World Congress on Intelligent Control and Automation.

[31] Buliali J.L., Hariadi V., Saikhu A., Mamase S. Generalized Regression Neural Network for predicting traffic flow. Proceedings of the 2016 International Conference on Information & Communication Technology and Systems (ICTS).

[32] Xiaobin L. RBF neural network optimized by particle swarm optimization for forecasting urban traffic flow. Proceedings of the 2009 Third International Symposium on Intelligent Information Technology Application.

[33] Zhu J.Z., Cao J.X., Zhu Y. (2014). Traffic volume forecasting based on radial basis function neural network with the consideration of traffic flows at the adjacent intersections. Transp. Res. Part C Emerg. Technol..

[34] Amin S.M., Rodin E., Liu A.P., Rink K., García-Ortiz A. (1998). Traffic prediction and management via RBF neural nets and semantic control. Comput. Aided Civ. Infrastruct. Eng..

[35] Ge Y., Wang G. (2011). Study of traffic flow short-time prediction based on wavelet neural network. Electrical Engineering and Control.

[36] Lin L., Li Y., Sadek A. (2013). A k nearest neighbor based local linear wavelet neural network model for on-line short-term traffic volume prediction. Procedia Soc. Behav. Sci..

[37] Li T., Sheng L. (2015). Prediction for short-term traffic flow based on optimized wavelet neural network model. Int. J. Comput. Sci. Inf. Technol..

[38] Yang H.j., Hu X. (2016). Wavelet neural network with improved genetic algorithm for traffic flow time series prediction. Optik.

[39] Lingras P., Mountford P. (2001). Time delay neural networks designed using genetic algorithms for short term inter-city traffic forecasting. International Conference on Industrial, Engineering and Other Applications of Applied Intelligent Systems.

[40] Zhong M., Sharma S., Lingras P. (2005). Short-Term Traffic Prediction on Different Types of Roads with Genetically Designed Regression and Time Delay Neural Network Models. J. Comput. Civ. Eng..

[41] Wang J., Tsapakis I., Zhong C. (2016). A space-time delay neural network model for travel time prediction. Eng. Appl. Artif. Intell..

[42] Khandani M.K., Mikhael W.B. Efficient Time Series Forecasting Using Time Delay Neural Networks with Domain Pre-Transforms. Proceedings of the 2019 IEEE 62nd International Midwest Symposium on Circuits and Systems (MWSCAS).

[43] Ulbricht C. (1994). Multi-Recurrent Networks for Traffic Forecasting.

[44] Yun S.Y., Namkoong S., Rho J.H., Shin S.W., Choi J.U. (1998). A performance evaluation of neural network models in traffic volume forecasting. Math. Comput. Model..

[45] Dia H. (2001). An object-oriented neural network approach to short-term traffic forecasting. Eur. J. Oper. Res..

[46] Ishak S., Kotha P., Alecsandru C. (2003). Optimization of dynamic neural network performance for short-term traffic prediction. Transp. Res. Rec..

[47] Zhang H. (2000). Recursive prediction of traffic conditions with neural network models. J. Transp. Eng..

[48] Bohan H., Yun B. Traffic Flow Prediction Based on BRNN. Proceedings of the 2019 IEEE 9th International Conference on Electronics Information and Emergency Communication (ICEIEC).

[49] Hochreiter S., Schmidhuber J. (1997). Long short-term memory. Neural Comput..

[50] Ma X., Tao Z., Wang Y., Yu H., Wang Y. (2015). Long short-term memory neural network for traffic speed prediction using remote microwave sensor data. Transp. Res. Part C Emerg. Technol..

[51] Khan Z., Khan S., Dey K., Chowdhury M. (2019). Development and Evaluation of Recurrent Neural Network-Based Models for Hourly Traffic Volume and Annual Average Daily Traffic Prediction. Transp. Res. Rec..

[52] Jia Y., Wu J., Ben-Akiva M., Seshadri R., Du Y. (2017). Rainfall-integrated traffic speed prediction using deep learning method. IET Intell. Transp. Syst..

[53] Zhao Z., Chen W., Wu X., Chen P.C., Liu J. (2017). LSTM network: A deep learning approach for short-term traffic forecast. IET Intell. Transp. Syst..

[54] Lu H., Ge Z., Song Y., Jiang D., Zhou T., Qin J. (2021). A temporal-aware LSTM enhanced by loss-switch mechanism for traffic flow forecasting. Neurocomputing.

[55] Chung J., Gulcehre C., Cho K., Bengio Y. (2015). Gated feedback recurrent neural networks. International Conference on Machine Learning.

[56] Fu R., Zhang Z., Li L. Using LSTM and GRU neural network methods for traffic flow prediction. Proceedings of the 2016 31st Youth Academic Annual Conference of Chinese Association of Automation (YAC).

[57] Varaiya P.P. (2007). Freeway Performance Measurement System (PeMS), PeMS 7.0.

[58] Zhao J., Gao Y., Qu Y., Yin H., Liu Y., Sun H. (2018). Travel time prediction: Based on gated recurrent unit method and data fusion. IEEE Access.

[59] Bartlett Z., Han L., Nguyen T., Johnson P. Prediction of road traffic flow based on deep recurrent neural networks. Proceedings of the 2019 IEEE SmartWorld, Ubiquitous Intelligence & Computing, Advanced & Trusted Computing, Scalable Computing & Communications, Cloud & Big Data Computing, Internet of People and Smart City Innovation (SmartWorld/SCALCOM/UIC/ATC/CBDCom/IOP/SCI).

[60] Pu Z., Cui Z., Wang S., Li Q., Wang Y. (2020). Time-aware gated recurrent unit networks for forecasting road surface friction using historical data with missing values. IET Intell. Transp. Syst..

[61] Khodabandelou G., Kheriji W., Selem F. (2021). Link traffic speed forecasting using convolutional attention-based gated recurrent unit. Appl. Intell..

[62] Ma X., Dai Z., He Z., Ma J., Wang Y., Wang Y. (2017). Learning traffic as images: A deep convolutional neural network for large-scale transportation network speed prediction. Sensors.

[63] Zang D., Wang D., Cheng J., Tang K., Li X. (2017). Traffic parameters prediction using a three-channel convolutional neural network. International Conference on Intelligence Science.

[64] Yu B., Yin H., Zhu Z. (2017). spatiotemporal graph convolutional networks: A deep learning framework for traffic forecasting. arXiv.

[65] Fouladgar M., Parchami M., Elmasri R., Ghaderi A. Scalable deep traffic flow neural networks for urban traffic congestion prediction. Proceedings of the 2017 International Joint Conference on Neural Networks (IJCNN).

[66] Hong H., Huang W., Song G., Xie K. (2014). Metric-based multitask grouping neural network for traffic flow forecasting. International Symposium on Neural Networks.

[67] Tan H., Xuan X., Wu Y., Zhong Z., Ran B. (2016). A comparison of traffic flow prediction methods based on DBN. CICTP 2016.

[68] Chen C., Wang H., Yuan F., Jia H., Yao B. (2020). Bus travel time prediction based on deep belief network with backpropagation. Neural Comput. Appl..

[69] Koesdwiady A., Soua R., Karray F. (2016). Improving traffic flow prediction with weather information in connected cars: A deep learning approach. IEEE Trans. Veh. Technol..

[70] Yin H., Wong S., Xu J., Wong C. (2002). Urban traffic flow prediction using a fuzzy-neural approach. Transp. Res. Part C Emerg. Technol..

[71] Quek C., Pasquier M., Lim B.B.S. (2006). POP-TRAFFIC: A novel fuzzy neural approach to road traffic analysis and prediction. IEEE Trans. Intell. Transp. Syst..

[72] Zhao L. (2012). Application of interval type–2 fuzzy neural networks to predict short–term traffic flow. Int. J. Comput. Appl. Technol..

[73] Li H. (2016). Research on prediction of traffic flow based on dynamic fuzzy neural networks. Neural Comput. Appl..

[74] Tang J., Liu F., Zou Y., Zhang W., Wang Y. (2017). An improved fuzzy neural network for traffic speed prediction considering periodic characteristic. IEEE Trans. Intell. Transp. Syst..

[75] An J., Fu L., Hu M., Chen W., Zhan J. (2019). A Novel Fuzzy-Based Convolutional Neural Network Method to Traffic Flow Prediction With Uncertain Traffic Accident Information. IEEE Access.

[76] Lv Y., Duan Y., Kang W., Li Z., Wang F.Y. (2014). Traffic flow prediction with big data: A deep learning approach. IEEE Trans. Intell. Transp. Syst..

[77] Yang H.F., Dillon T.S., Chen Y.P.P. (2016). Optimized structure of the traffic flow forecasting model with a deep learning approach. IEEE Trans. Neural Netw. Learn. Syst..

[78] Xiang J., Chen Z. (2018). Traffic State Estimation of Signalized Intersections Based on Stacked Denoising Auto-Encoder Model. Wirel. Pers. Commun..

[79] Park D., Rilett L.R. (1998). Forecasting Multiple-Period Freeway Link Travel Times Using Modular Neural Networks. Transp. Res. Rec. J. Transp. Res. Board.

[80] Ishak S., Alecsandru C. (2004). Optimizing traffic prediction performance of neural networks under various topological, input, and traffic condition settings. J. Transp. Eng..

[81] Vlahogianni E.I., Karlaftis M.G., Golias J.C. (2007). spatiotemporal short-term urban traffic volume forecasting using genetically optimized modular networks. Comput. Aided Civ. Infrastruct. Eng..

[82] Tung W., Quek C. (2002). GenSoFNN: A generic self-organizing fuzzy neural network. IEEE Trans. Neural Netw..

[83] Boto-Giralda D., Díaz-Pernas F.J., González-Ortega D., Díez-Higuera J.F., Antón-Rodríguez M., Martínez-Zarzuela M., Torre-Díez I. (2010). Wavelet-based denoising for traffic volume time series forecasting with self-organizing neural networks. Comput. Aided Civ. Infrastruct. Eng..

[84] Ll J., Huang J. (2016). Prediction of network traffic using local autoregressive methods based on self-organizing map neural network. Inf. Control.

[85] Petridis V., Kehagias A., Petrou L., Bakirtzis A., Kiartzis S., Panagiotou H., Maslaris N. (2001). A Bayesian Multiple Models Combination Method for Time Series Prediction. J. Intell. Robot. Syst..

[86] Chan K.Y., Dillon T., Chang E., Singh J. (2012). Prediction of short-term traffic variables using intelligent swarm-based neural networks. IEEE Trans. Control Syst. Technol..

[87] Gu Y., Lu W., Xu X., Qin L., Shao Z., Zhang H. (2020). An improved bayesian combination model for short-term traffic prediction with deep learning. IEEE Trans. Intell. Transp. Syst..

[88] AlKheder S., Alkhamees W., Almutairi R., Alkhedher M. (2021). Bayesian combined neural network for traffic volume short-term forecasting at adjacent intersections. Neural Comput. Appl..

[89] Chen H., Grant-Muller S. (2001). Use of sequential learning for short-term traffic flow forecasting. Transp. Res. Part C Emerg. Technol..

[90] Bouyahia Z., Haddad H., Jabeur N., Yasar A. (2019). A two-stage road traffic congestion prediction and resource dispatching toward a self-organizing traffic control system. Pers. Ubiquitous Comput..

[91] Cui Y., Huang X., Wu D., Zheng H. (2020). Machine Learning-Based Resource Allocation Strategy for Network Slicing in Vehicular Networks. Wirel. Commun. Mob. Comput..

[92] Goodfellow I.J., Pouget-Abadie J., Mirza M., Xu B., Warde-Farley D., Ozair S., Courville A., Bengio Y. (2014). Generative Adversarial Networks. arXiv.

[93] Liang Y., Cui Z., Tian Y., Chen H., Wang Y. (2018). A deep generative adversarial architecture for network-wide spatial-temporal traffic-state estimation. Transp. Res. Rec..

[94] Zhang Y., Wang S., Chen B., Cao J., Huang Z. (2021). TrafficGAN: Network-Scale Deep Traffic Prediction With Generative Adversarial Nets. IEEE Trans. Intell. Transp. Syst..

[95] Zhang L., Wu J., Shen J., Chen M., Wang R., Zhou X., Xu C., Yao Q., Wu Q. (2021). SATP-GAN: Self-attention based generative adversarial network for traffic flow prediction. Transp. B.

[96] Li X., Rosman G., Gilitschenski I., Vasile C.I., DeCastro J.A., Karaman S., Rus D. (2021). Vehicle Trajectory Prediction Using Generative Adversarial Network With Temporal Logic Syntax Tree Features. IEEE Robot. Autom. Lett..

[97] Yang H., Li X., Qiang W., Zhao Y., Zhang W., Tang C. (2021). A network traffic forecasting method based on SA optimized ARIMA-BP neural network. Comput. Netw..

[98] Lin X., Huang Y. (2021). Short-Term High-Speed Traffic Flow Prediction Based on ARIMA-GARCH-M Model. Wirel. Pers. Commun..

[99] Ding C., Duan J., Zhang Y., Wu X., Yu G. (2017). Using an ARIMA-GARCH modeling approach to improve subway short-term ridership forecasting accounting for dynamic volatility. IEEE Trans. Intell. Transp. Syst..

[100] Luo X., Li D., Yang Y., Zhang S. (2019). Spatiotemporal traffic flow prediction with KNN and LSTM. J. Adv. Transp..

[101] Li Y., Yu R., Shahabi C., Liu Y. (2017). Diffusion convolutional recurrent neural network: Data-driven traffic forecasting. arXiv.

[102] Yu H., Wu Z., Wang S., Wang Y., Ma X. (2017). Spatiotemporal recurrent convolutional networks for traffic prediction in transportation networks. Sensors.

[103] Allström A., Ekström J., Gundlegård D., Ringdahl R., Rydergren C., Bayen A.M., Patire A.D. (2016). Hybrid approach for short-term traffic state and travel time prediction on highways. Transp. Res. Rec..

[104] Kolidakis S., Botzoris G., Profillidis V., Lemonakis P. (2019). Road traffic forecasting—A hybrid approach combining artificial neural network with singular spectrum analysis. Econ. Anal. Policy.

[105] Hussain A., Wang T., Cao J. (2020). Optimizing Traffic Lights with Multi-agent Deep Reinforcement Learning and V2X communication. arXiv.

[106] Hale D. (2005). Traffic Network Study Tool–TRANSYT-7F, United States Version.

[107] Zheng M., Xu H., Zhang K., Yao R. (2017). Shortest-way: An improved empirical transition method for signal coordination. J. Adv. Transp..

[108] Zhang L., Yin Y., Lou Y. (2010). Robust signal timing for arterials under day-to-day demand variations. Transp. Res. Rec..

[109] Jang K., Kim H., Jang I.G. (2015). Traffic signal optimization for oversaturated urban networks: Queue growth equalization. IEEE Trans. Intell. Transp. Syst..

[110] Mohajerpoor R., Saberi M., Ramezani M. (2019). Analytical derivation of the optimal traffic signal timing: Minimizing delay variability and spillback probability for undersaturated intersections. Transp. Res. Part B Methodol..

[111] Webster F.V. (1958). Traffic Signal Settings.

[112] Webster F.V., Cobbe B.M., Road Research Laboratory (1966). Traffic Signals.

[113] Kouvelas A., Aboudolas K., Papageorgiou M., Kosmatopoulos E.B. (2011). A hybrid strategy for real-time traffic signal control of urban road networks. IEEE Trans. Intell. Transp. Syst..

[114] Ali M.E.M., Durdu A., Celtek S.A., Gultekin S.S. (2020). Fuzzy Logic and Webster’s Optimal Cycle Based Decentralized Coordinated Adaptive Traffic Control Method. Elektron. Elektrotechnika.

[115] Calle-Laguna A.J., Du J., Rakha H.A. (2019). Computing optimum traffic signal cycle length considering vehicle delay and fuel consumption. Transp. Res. Interdiscip. Perspect..

[116] Bretherton R. (1990). SCOOT urban traffic control system—Philosophy and evaluation. IFAC Proc. Vol..

[117] Hansen B.G., Martin P.T., Joseph Perrin H. (2000). SCOOT real-time adaptive control in a CORSIM simulation environment. Transp. Res. Rec..

[118] Mauro V., Di Taranto C. (1990). Utopia. IFAC Proc. Vol..

[119] Wahlstedt J. Evaluation of the two self-optimising traffic signal systems Utopia/Spot and ImFlow, and comparison with existing signal control in Stockholm, Sweden. Proceedings of the 16th International IEEE Conference on Intelligent Transportation Systems (ITSC 2013).

[120] Pavleski D., Koltovska-Nechoska D., Ivanjko E. Evaluation of adaptive traffic control system UTOPIA using microscopic simulation. Proceedings of the 2017 International Symposium ELMAR.

[121] Tassiulas L., Ephremides A. Stability properties of constrained queueing systems and scheduling policies for maximum throughput in multihop radio networks. Proceedings of the 29th IEEE Conference on Decision and Control.

[122] Varaiya P. (2013). Max pressure control of a network of signalized intersections. Transp. Res. Part C Emerg. Technol..

[123] Le T., Kovács P., Walton N., Vu H.L., Andrew L.L., Hoogendoorn S.S. (2015). Decentralized signal control for urban road networks. Transp. Res. Part C Emerg. Technol..

[124] Gregoire J., Qian X., Frazzoli E., De La Fortelle A., Wongpiromsarn T. (2014). Capacity-aware backpressure traffic signal control. IEEE Trans. Control Netw. Syst..

[125] Zaidi A.A., Kulcsár B., Wymeersch H. (2016). Back-pressure traffic signal control with fixed and adaptive routing for urban vehicular networks. IEEE Trans. Intell. Transp. Syst..

[126] Levin M.W., Boyles S.D. (2017). Pressure-Based Policies for Reservation-Based Intersection Control.

[127] Nilsson G., Como G. (2017). On generalized proportional allocation policies for traffic signal control. IFAC-PapersOnLine.

[128] Nilsson G., Como G. (2020). Generalized proportional allocation policies for robust control of dynamical flow networks. IEEE Trans. Autom. Control.

[129] Nilsson G., Como G. (2019). A micro-simulation study of the generalized proportional allocation traffic signal control. IEEE Trans. Intell. Transp. Syst..

[130] Nilsson G., Como G. On Robustness of the Generalized Proportional Controller for Traffic Signal Control. Proceedings of the 2020 American Control Conference (ACC).

[131] Smith M. (1993). The interactions between signal control policies and route choice. Transportation and Traffic Theory.

[132] Cantelmo G., Viti F., Rinaldi M., Tampère C.M., Smith M.J., Nökel K. Systematic assessment of local & global signal control policies: A methodological perspective. Proceedings of the 2015 International Conference on Models and Technologies for Intelligent Transportation Systems (MT-ITS).

[133] Smith M.J., Iryo T., Mounce R., Rinaldi M., Viti F. (2019). Traffic control which maximises network throughput: Some simple examples. Transp. Res. Part C Emerg. Technol..

[134] Abdulhai B., Pringle R., Karakoulas G.J. (2003). Reinforcement learning for true adaptive traffic signal control. J. Transp. Eng..

[135] Wiering M., Veenen J.v., Vreeken J., Koopman A. (2004). Intelligent Traffic Light Control.

[136] Wunderlich R., Liu C., Elhanany I., UrbanikII T. (2008). A novel signal-scheduling algorithm with quality-of-service provisioning for an isolated intersection. IEEE Trans. Intell. Transp. Syst..

[137] Arel I., Liu C., Urbanik T., Kohls A.G. (2010). Reinforcement learning-based multi-agent system for network traffic signal control. IET Intell. Transp. Syst..

[138] Prashanth L., Bhatnagar S. Reinforcement learning with average cost for adaptive control of traffic lights at intersections. Proceedings of the 2011 14th International IEEE Conference on Intelligent Transportation Systems (ITSC).

[139] Abdoos M., Mozayani N., Bazzan A.L. Traffic light control in non-stationary environments based on multi agent Q-learning. Proceedings of the 2011 14th International IEEE Conference on Intelligent Transportation Systems (ITSC).

[140] Abdoos M., Mozayani N., Bazzan A.L. (2013). Holonic multi-agent system for traffic signals control. Eng. Appl. Artif. Intell..

[141] Aziz H.A., Zhu F., Ukkusuri S.V. (2018). Learning-based traffic signal control algorithms with neighborhood information sharing: An application for sustainable mobility. J. Intell. Transp. Syst..

[142] Genders W., Razavi S. (2019). Asynchronous n-step Q-learning adaptive traffic signal control. J. Intell. Transp. Syst..

[143] Hua J., Faghri A. (1995). Development of Neural Signal Control System—Toward Intelligent Traffic Signal Control.

[144] Spall J.C., Chin D.C. (1997). Traffic-responsive signal timing for system-wide traffic control. Transp. Res. Part C Emerg. Technol..

[145] Saito M., Fan J. (2000). Artificial neural network–based heuristic optimal traffic signal timing. Comput. Aided Civ. Infrastruct. Eng..

[146] Kim J.T., Lee J., Chang M. (2008). Neural-network-based cycle length design for real-time traffic control. Can. J. Civ. Eng..

[147] Jeon H., Lee J., Sohn K. (2018). Artificial intelligence for traffic signal control based solely on video images. J. Intell. Transp. Syst..

[148] Bernas M., Płaczek B., Smyła J. (2019). A Neuroevolutionary Approach to Controlling Traffic Signals Based on Data from Sensor Network. Sensors.

[149] Mir A., Hassan A. Fuzzy Inference Rule based Neural Traffic Light Controller. Proceedings of the 2018 IEEE International Conference on Mechatronics and Automation (ICMA).

[150] Dong C., Yang K., Guo J., Chen X., Dong H., Bai Y. Analysis and Control of Intelligent Traffic Signal System Based on Adaptive Fuzzy Neural Network. Proceedings of the 2019 5th International Conference on Transportation Information and Safety (ICTIS).

[151] Mittal U., Chawla P. Neuro—Fuzzy Based Adaptive Traffic Light Management system. Proceedings of the 2020 8th International Conference on Reliability, Infocom Technologies and Optimization (Trends and Future Directions) (ICRITO).

[152] Chen P., Zhu Z., Lu G. An Adaptive Control Method for Arterial Signal Coordination Based on Deep Reinforcement Learning*. Proceedings of the 2019 IEEE Intelligent Transportation Systems Conference (ITSC).

[153] Luo J., Li X., Zheng Y. Researches on intelligent traffic signal control based on deep reinforcement learning. Proceedings of the 2020 16th International Conference on Mobility, Sensing and Networking (MSN).

[154] Li Z., Yu H., Zhang G., Dong S., Xu C.Z. (2021). Network-wide traffic signal control optimization using a multi-agent deep reinforcement learning. Transp. Res. Part C Emerg. Technol..

[155] Wang T., Cao J., Hussain A. (2021). Adaptive Traffic Signal Control for large-scale scenario with Cooperative Group-based Multi-agent reinforcement learning. Transp. Res. Part C Emerg. Technol..

[156] Kekuda A., Anirudh R., Krishnan M. Reinforcement Learning based Intelligent Traffic Signal Control using n-step SARSA. Proceedings of the 2021 International Conference on Artificial Intelligence and Smart Systems (ICAIS).

[157] Zhao X.H., Li Z.L., Chen Y.Z. (2006). Hybrid control based on Q-learning for urban traffic signal. J. Syst. Simul..

[158] Pálos P., Huszák Á. Comparison of Q-Learning based Traffic Light Control Methods and Objective Functions. Proceedings of the 2020 International Conference on Software, Telecommunications and Computer Networks (SoftCOM).

[159] Dampage S., Munasingha T., Gunathilake W., Weerasundara A., Udugahapattuwa D. Adaptive & Coordinated Traffic Signal System. Proceedings of the 2020 IEEE International Conference on Computing, Power and Communication Technologies (GUCON).

[160] Mao T., Mihaita A., Chen F., Vu H. (2021). Boosted Genetic Algorithm Using Machine Learning for Traffic Control Optimization. IEEE Trans. Intell. Transp. Syst..

[161] Maghazeh A., Bordoloi U.D., Eles P., Peng Z. General purpose computing on low-power embedded GPUs: Has it come of age?. Proceedings of the 2013 International Conference on Embedded Computer Systems: Architectures, Modeling, and Simulation (SAMOS).

[162] Duan Q. (2017). Cloud service performance evaluation: Status, challenges, and opportunities—A survey from the system modeling perspective. Digit. Commun. Netw..

[163] Strubell E., Ganesh A., McCallum A. (2019). Energy and policy considerations for deep learning in NLP. arXiv.

